# Epidemiological Transitions in Breast Cancer Burden across Socioeconomic Settings, 1990–2021: An Ecological Time‐Trend Study of China, the United Kingdom, and India

**DOI:** 10.1002/hsr2.73479

**Published:** 2026-11-10

**Authors:** Zhangyue Sun, Xiaolu Tan, Qianrui Xu, Hong Liang, Jie Chen

**Affiliations:** ^1^ Department of Medical Oncology, The Third Affiliated Hospital Sun Yat‐sen University Guangzhou China; ^2^ Shantou University Medical College Shantou China; ^3^ Department of Breast Surgery Shanghai Sixth People's Hospital Affiliated to Shanghai Jiao Tong University School of Medicine Shanghai China; ^4^ University of Heidelberg Heidelberg Germany

**Keywords:** breast cancer, DALYs, epidemiological transition, global burden, metabolic risks, socioeconomic disparities

## Abstract

**Background and Aims:**

Breast cancer (BC) remains the most common malignancy among women worldwide and a leading cause of cancer‐related mortality. As countries undergo demographic, socioeconomic, and lifestyle transitions, the burden of BC and its attributable risk factors continue to evolve. This study aimed to characterize global and regional trends in BC burden from 1990 to 2021 and compared epidemiological patterns across different socioeconomic settings.

**Methods:**

Data were obtained from the Global Burden of Disease (GBD) 2021 study to assess BC incidence, mortality, disability‐adjusted life years (DALYs), and attributable risk factors by age, sex, and sociodemographic index (SDI). GLOBOCAN 2022 esitimates were used as an independent contemporary comparison of cross‐national burden patterns. Temporal trends were quantified using estimated annual percentage change (EAPC). China, the United Kingdom (UK), and India were selected as representative countries with distinct socioeconomic profiles.

**Results:**

Globally, the age‐standardized incidence rate (ASIR) of BC increased (EAPC = 0.38, 95% CI 0.33–0.42), whereas the age‐standardized death rate (ASDR) declined (EAPC = −0.59, 95% CI −0.64 to −0.54) between 1990 and 2021. High‐SDI regions and the UK experienced reductions in both incidence and mortality, while China showed increasing incidence but decreasing mortality, consistent with an epidemiological transition pattern. In contrast, India exhibited concurrent increases in incidence and mortality. GLOBOCAN 2022 estimates showed similar broad cross‐national heterogeneity, although the absolute age‐standardized rates differed substantially from the corresponding GBD estimates. Age‐specific DALY distributions differed markedly across countries, with aging‐related burden predominating in the UK, middle‐aged burden in China, and younger burden in India. Furthermore, the contribution of behavioral risk factors became increasingly important.

**Conclusion:**

The burden of BC demonstrates substantial heterogeneity across socioeconomic settings. Distinct epidemiological transition patterns and evolving risk factor profiles highlight the need for region‐specific prevention strategies, metabolic risk management, early detection programs, and equitable access to healthcare services.

**Abbreviations and Acronyms**
ASIR: Age‐Standardized Incidence Rate
ASDR: Age‐Standardized Death Rate
BC: Breast Cancer
DALY: Disability‐Adjusted Life Year
EAPC: Estimated Annual Percentage Change
FPG: Fasting Plasma Glucose
GBD: Global Burden of Disease
GBD regions: Global Burden of Disease regions (21 geographic regions defined in GBD study)
LMICs: Low‐ and Middle‐Income Countries
SDI: Socio‐demographic Index
UI: Uncertainty Interval
YLD: Years Lived with Disability
YLL: Years of Life Lost

## 1 Introduction

Breast cancer (BC) has become the most commonly diagnosed cancer and the leading cause of cancer‐related mortality among women worldwide, with an estimated 2.3 million new cases and 665,684 deaths in 2022, accounting for 23.8% of all cancer diagnoses [1]. Despite global improvements in cancer control, substantial geographical disparities remain: high‐SDI regions report higher incidence but lower mortality due to widespread screening and effective treatment, whereas many low‐ and middle‐income countries (LMICs) face rising incidence, limited awareness, delayed diagnosis, and poorer survival outcomes [2, 3]. These disparities reflect ongoing epidemiological transitions associated with population aging, westernized lifestyles, metabolic risk accumulation, and unequal healthcare access across different socioeconomic settings [4]. Without coordinated global efforts, these inequalities in breast cancer burden are likely to widen further.

Although previous GBD‐based studies have described the global or regional burden of BC, most have focused primarily on temporal trends or individual risk factors. Less attention has been paid to how breast cancer burden transitions across socioeconomic settings, particularly regarding age‐structured burden patterns and the shift from behavioral to metabolic risk factors [5, 6, 7, 8, 9, 10]. Understanding these epidemiological transitions is essential for developing region‐specific prevention strategies and reducing global disparities in BC outcomes.

Therefore, this study aimed to provide a comprehensive assessment of the global BC burden using GBD 2021 data, with particular emphasis on epidemiological transitions across socioeconomic settings. We analyzed incidence, mortality, and DALYs from 1990 to 2021 by age, sex, and sociodemographic index (SDI) level, and further evaluated the contribution of six major modifiable risk factors, including diet high in red meat, alcohol use, high BMI, high fasting plasma glucose (FPG), low physical activity, and tobacco use.

To illustrate distinct transition patterns, we further conducted in‐depth analyses of China, the United Kingdom, and India as representative profiles of high‐middle, high, and low‐middle SDI settings, respectively, according to the GBD 2021 classification [11]. These countries represent different stages of BC burden transition, ranging from aging‐associated survivorship patterns to emerging metabolic‐risk transitions and persistent early‐onset burden [2, 12]. By integrating global trends with national transition profiles, our study provides insights into the evolving epidemiology of BC and informs tailored prevention strategies across different socioeconomic contexts.

## 2 Methods

### 2.1 Data Source

Annual incident cases, deaths, age‐standardized incidence rates (ASIRs), age‐standardized death rates (ASDRs), and disability‐adjusted life years (DALYs) of breast cancer from 1990 to 2021 were obtained from the Global Burden of Disease (GBD) 2021 study through the Global Health Data Exchange (GHDx) query tool. Data were extracted by sex, age, region, country, the 21 geographic regions defined by the GBD study, and the five Socio‐demographic Index (SDI) categories. SDI is a composite measure based on lag‐distributed income per capita, mean educational attainment among individuals aged ≥ 15 years, and the total fertility rate among individuals aged < 25 years.

The attributable risk factors evaluated in this study included four behavioral risks—diet high in red meat, alcohol use, tobacco use, and low physical activity—and two metabolic risks—high body‐mass index (BMI) and high fasting plasma glucose (FPG). The methodology of the GBD 2021 study has been described elsewhere [11, 13, 14]. To provide an independent contemporary comparison of the cross‐national patterns, BC incidence and mortality estimate for 2022 were obtained from the GLOBOCAN 2022 database developed by the International Agency for Research on Cancer. GBD and GLOBOCAN differ in their reference years, input data sources, estimation and extrapolation procedures, and age‐standardization methods; therefore, their absolute estimates were not considered directly interchangeable [15, 16]. Ethics approval and informed consent were not required for this study because of public accessibility of the data. This study followed the Guidelines for Accurate and Transparent Health Estimates Reporting (GATHER) for cross‐sectional studies.

### 2.2 Statistical Analysis

#### 2.2.1 Temporal Trend Analysis

Age‐standardized rates (ASRs), including the age‐standardized incidence rate (ASIR), age‐standardized death rate (ASDR), and age‐standardized disability‐adjusted life‐year (DALY) rate, were expressed per 100,000 population. Estimates derived from the Global Burden of Disease (GBD) 2021 study are presented with 95% uncertainty intervals (UIs). Temporal trends from 1990 to 2021 were quantified using the estimated annual percentage change (EAPC). The EAPC was estimated by fitting a log‐linear regression model to the age‐standardized rate over calendar year:

ln (ASR) = α + βx + ε

where x represents the calendar year, α is the intercept, β is the regression coefficient for calendar year, and εis the error term. The EAPC was computed as:

100 × (e*β* − 1)

An EAPC with both limits of the 95% confidence interval (CI) above zero indicated an increasing trend, whereas values below zero indicated a decreasing trend.

#### 2.2.2 Age‐Structured and Risk‐Attributable Analyses

Age‐ and sex‐specific distributions of DALYs were analyzed to characterize demographic transition patterns of breast cancer burden across different socioeconomic settings. In addition, six major modifiable risk factors, including diet high in red meat, alcohol use, high body‐mass index (BMI), high fasting plasma glucose (FPG), low physical activity, and tobacco use, were evaluated to assess transitions in risk‐attributable burden patterns. Risk‐attributable proportions were calculated as the estimated DALYs attributable to a specific risk factor divided by the estimated total breast cancer DALYs for the corresponding location, year, sex, and age category. Because attributable burdens across individual risk factors may overlap, these proportions were not summed or interpreted as mutually exclusive components of the total breast cancer burden.

#### 2.2.3 MIR Analysis

The mortality‐to‐incidence ratio (MIR) was used as an ecological, rate‐based indicator of mortality relative to incidence. For the GBD 2021 estimates, MIR was calculated by dividing the age‐standardized death rate (ASDR) by the age‐standardized incidence rate (ASIR) for the same location and year. For GLOBOCAN 2022, MIR was calculated using the corresponding ASDR and ASIR. For consistency, the age‐standardized mortality rate terminology used is referred to as ASDR throughout this study. MIR was used only for descriptive cross‐national comparisons and was not interpreted as a direct measure of patient survival or healthcare accessibility.

#### 2.2.4 Statistical Software

All analyses were conducted using R software (version 4.3.1; R Foundation for Statistical Computing, Vienna, Austria) [17]. Statistical inference for temporal trends was based on the two‐sided 95% CI of the EAPC rather than on separately reported P values. The age‐, sex‐, SDI‐, risk factor‐, and country‐specific comparisons were descriptive and exploratory. Because the study was based on aggregated population‐level estimates, the analyses were not intended to establish individual‐level or causal associations. Statistical reporting followed the Statistical Analyses and Methods in the Published Literature (SAMPL) guidelines [18].

## 3 Results

### 3.1 Global and Socioeconomic Transition Patterns of Breast Cancer Burden (1990–2021)

Globally, the age‐standardized incidence rate (ASIR) of breast cancer increased modestly from 1990 to 2021 (EAPC = 0.38; 95% CI: 0.33–0.42), whereas the age‐standardized death rate (ASDR) declined steadily (EAPC = −0.59; 95% CI: −0.64 to −0.54) (Figure 1). By Socio‐demographic Index (SDI), divergent patterns were observed. High‐SDI regions exhibited decreases in both ASIR (EAPC = −0.27; 95% CI: −0.39 to −0.16) and ASDR (EAPC = −1.62; 95% CI: −1.66 to −1.57). In high‐middle SDI regions, ASIR continued to rise (EAPC = 0.76; 95% CI: 0.69–0.83), while ASDR decreased (EAPC = −0.92; 95% CI: −1.02 to −0.82). In contrast, both ASIR and ASDR increased in low‐middle regions (ASIR: EAPC, 2.27; 95% CI, 2.23–2.31; ASDR: EAPC, 1.37; 95% CI, 1.32–1.41) and low SDI regions (ASIR: EAPC, 1.39; 95% CI, 1.25–1.41; ASDR: EAPC, 0.88; 95% CI, 0.76–1). Table 1 summarizes the global burden of BC in 1990 and 2021.

**Figure 1:**
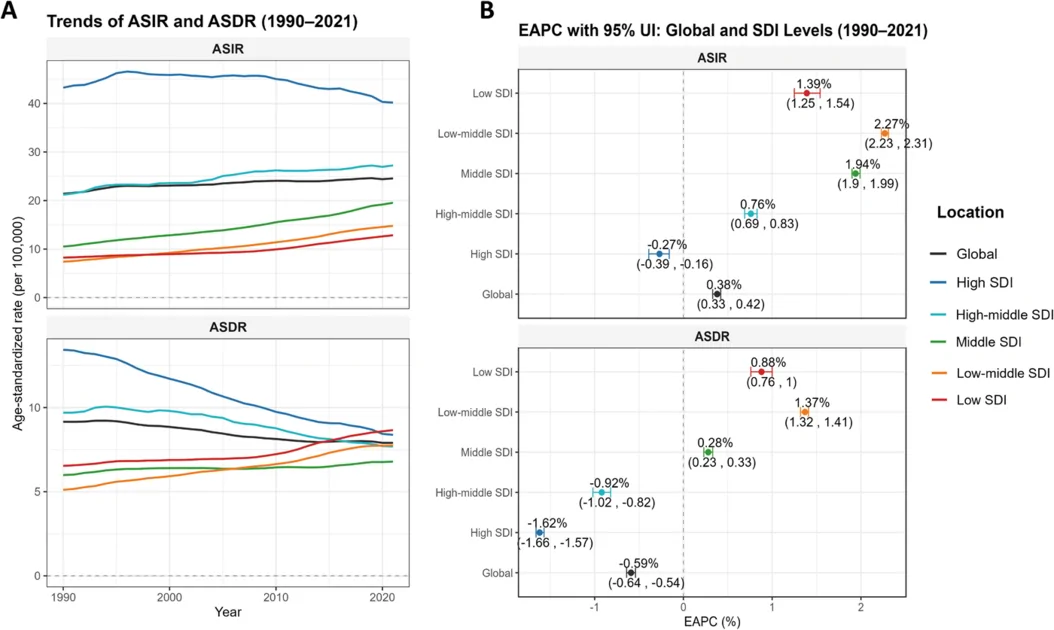
Global and socioeconomic transition patterns of breast cancer incidence and mortality burden, 1990–2021. (A) show temporal trends in age‐standardized incidence rate (ASIR, upper) and age‐standardized death rate (ASDR, lower) of breast cancer across the global population and five Socio‐demographic Index (SDI) regions from 1990 to 2021. (B) display the EAPC with corresponding 95% uncertainty intervals (UI) for ASIR (upper) and ASDR (lower). Positive EAPC values indicate increasing trends, whereas negative values indicate decreasing trends.

**Table 1:** Global and region‐specific burden of breast cancer in 1990 and 2021, with estimated annual percentage changes (EAPCs) during 1990–2021.

Location	1990				2021				1990–2021	
	Incidence×10^3^ (95%UI)	ASIR per 100,000 (95%UI)	Death×10^3^ (95%UI)	ASDR per100,000 (95%UI)	Incidence×10^3^ (95%UI)	ASIR per 100,000 (95%UI)	Death×10^3^ (95%UI)	ASDR per 100,000 (95%UI)	EAPC of ASIR (95%UI)	EAPC of ASDR (95%UI)
Global	875.66 (834.23, 910.53)	21.38 (20.27, 22.22)	355.48 (335.47, 373.48)	9.16 (8.57, 9.61)	2121.56 (1982.14, 2268.72)	24.56 (22.93, 26.26)	674.2 (623.37, 720.82)	7.9 (7.27, 8.44)	0.38% (0.33, 0.42)	−0.59% (−0.64, −0.54)
Sex										
Male	9.78 (8.47, 11.22)	0.52 (0.46, 0.6)	4.9 (4.14, 5.9)	0.28 (0.24, 0.34)	38.83 (24.65, 47.85)	0.94 (0.6, 1.15)	13.27 (9.07, 16.24)	0.34 (0.23, 0.41)	2.21% (2.05, 2.37)	0.66% (0.57, 0.76)
Female	865.88 (824.34, 900.79)	39.99 (38.01, 41.6)	350.58 (330.51, 368.43)	16.6 (15.6, 17.45)	2082.74 (1940.35, 2225.08)	46.4 (43.26, 49.56)	660.93 (609.17, 707.18)	14.55 (13.45, 15.56)	0.4% (0.35, 0.45)	−0.55% (−0.6, −0.5)
SDI										
Low SDI	21.28 (18.2, 24.81)	8.23 (7.08, 9.47)	15.71 (13.46, 18.27)	6.53 (5.64, 7.5)	78.13 (68.9, 87.59)	12.84 (11.35, 14.3)	47.44 (41.89, 53.33)	8.66 (7.66, 9.66)	1.39% (1.25, 1.54)	0.88% (0.76, 1)
Low‐middle SDI	53.16 (47.76, 60.12)	7.39 (6.64, 8.31)	34.42 (30.7, 39.03)	5.11 (4.55, 5.76)	239.54 (216.75, 261.14)	14.78 (13.36, 16.1)	118.46 (106.61, 129.67)	7.75 (6.96, 8.5)	2.27% (2.23, 2.31)	1.37% (1.32, 1.41)
Middle SDI	125.57 (115.59, 137.9)	10.52 (9.7, 11.49)	65.6 (60.4, 72.17)	5.99 (5.53, 6.53)	552.88 (499.16, 612.2)	19.54 (17.67, 21.62)	185.77 (168.49, 206.08)	6.79 (6.16, 7.54)	1.94% (1.9, 1.99)	0.28% (0.23, 0.33)
High‐middle SDI	215.08 (204.49, 225.12)	21.19 (20.14, 22.16)	94.37 (89.27, 98.92)	9.7 (9.14, 10.17)	517.07 (468.47, 576.71)	27.24 (24.69, 30.42)	148.44 (134.92, 161.65)	7.68 (6.98, 8.37)	0.76% (0.69, 0.83)	−0.92% (−1.02, −0.82)
High SDI	459.47 (438.29, 470.35)	43.25 (41.36, 44.25)	144.87 (136.61, 149.55)	13.44 (12.69, 13.87)	731.76 (668.8, 764.12)	40.17 (37.29, 41.71)	173.26 (153.06, 183.93)	8.38 (7.55, 8.82)	−0.27% (−0.39, −0.16)	−1.62% (−1.66, −1.57)
Region										
High‐income Asia Pacific	31.39 (29.72, 32.94)	15.18 (14.37, 15.91)	7.83 (7.47, 8.08)	3.84 (3.66, 3.97)	97.15 (85.26, 105.26)	28.44 (25.74, 30.43)	20.23 (16.85, 22.17)	4.89 (4.32, 5.23)	2.13% (1.89, 2.37)	0.79% (0.65, 0.93)
High‐income North America	221.97 (210.89, 228.57)	66.89 (63.79, 68.76)	54.02 (50.56, 56.03)	15.85 (14.89, 16.39)	298.02 (274.48, 311.95)	50.57 (47.29, 52.74)	59.59 (53.31, 63.19)	9.32 (8.46, 9.84)	−1.08% (−1.18, −0.98)	−1.86% (−1.91, −1.8)
Western Europe	237.56 (226.21, 244.51)	45.27 (43.27, 46.51)	91.97 (86.19, 95.31)	16.64 (15.66, 17.2)	335.27 (302.44, 355.61)	43.09 (40.06, 45.4)	93.88 (80.68, 101.17)	9.93 (8.82, 10.57)	−0.13% (−0.31, 0.05)	−1.73% (−1.78, −1.69)
Australasia	9.9 (9.37, 10.43)	43.53 (41.28, 45.77)	3.29 (3.08, 3.48)	14.49 (13.56, 15.34)	19.85 (17.62, 22.08)	42.36 (37.94, 47.09)	4.37 (3.75, 4.93)	8.34 (7.31, 9.29)	−0.11% (−0.29, 0.06)	−1.85% (−1.91, −1.79)
Andean Latin America	2.36 (2.02, 2.73)	10.2 (8.76, 11.79)	1.38 (1.18, 1.6)	6.32 (5.39, 7.33)	10.9 (8.54, 13.86)	17.51 (13.73, 22.33)	4.12 (3.26, 5.2)	6.82 (5.39, 8.62)	1.54% (1.4, 1.69)	0.01% (−0.12, 0.13)
Tropical Latin America	16.31 (15.69, 16.92)	15.9 (15.23, 16.52)	8.53 (8.18, 8.85)	8.99 (8.51, 9.37)	62.5 (58.53, 66.09)	23.75 (22.21, 25.13)	24.42 (22.6, 25.92)	9.43 (8.71, 10.03)	1.02% (0.92, 1.12)	−0.02% (−0.09, 0.04)
Central Latin America	15.03 (14.56, 15.45)	15.43 (14.94, 15.86)	5.65 (5.48, 5.79)	6.35 (6.12, 6.52)	77.2 (67.26, 87.27)	29.39 (25.65, 33.2)	19.49 (17.03, 21.94)	7.63 (6.67, 8.58)	1.87% (1.77, 1.98)	0.47% (0.37, 0.57)
Southern Latin America	12.15 (11.48, 12.77)	26.28 (24.78, 27.59)	7.17 (6.75, 7.51)	15.81 (14.84, 16.63)	22.97 (21.14, 24.78)	27.45 (25.29, 29.6)	10.08 (9.08, 10.94)	11.64 (10.53, 12.59)	0.17% (0.02, 0.32)	−0.93% (−1.06, −0.8)
Caribbean	6.07 (5.68, 6.5)	22.55 (21.13, 24.08)	2.64 (2.43, 2.88)	10.19 (9.42, 11.08)	15.18 (12.9, 17.38)	28.46 (24.17, 32.62)	5.69 (4.81, 6.68)	10.58 (8.94, 12.43)	0.82% (0.73, 0.91)	0.22% (0.15, 0.28)
Central Europe	36.4 (34.94, 37.89)	24.87 (23.83, 25.87)	17.65 (16.92, 18.41)	12.24 (11.71, 12.79)	65.49 (60.23, 70.8)	33.17 (30.56, 35.96)	25.22 (22.97, 27.34)	11.61 (10.64, 12.58)	0.85% (0.7, 1.01)	−0.35% (−0.45, −0.25)
Eastern Europe	65.3 (63.26, 67.23)	23.99 (23.22, 24.69)	30.03 (29.06, 30.87)	11 (10.64, 11.3)	101.09 (90.96, 112.24)	30.6 (27.53, 34.02)	36.12 (32.26, 40.81)	10.54 (9.41, 11.94)	0.49% (0.37, 0.61)	−0.55% (−0.78, −0.33)
Central Asia	9.83 (9.25, 10.33)	19.75 (18.59, 20.78)	5.17 (4.87, 5.44)	10.74 (10.12, 11.3)	15.44 (13.78, 17.28)	16.91 (15.16, 18.85)	6.65 (5.95, 7.42)	7.78 (6.99, 8.63)	−0.22% (−0.3, −0.14)	−0.75% (−0.84, −0.66)
North Africa and Middle East	16.58 (14.8, 18.93)	8.19 (7.37, 9.27)	6.98 (6.27, 7.95)	3.76 (3.39, 4.23)	128.35 (114.49, 144.65)	23.74 (21.16, 26.69)	30.14 (26.53, 34.26)	6.11 (5.41, 6.92)	3.97% (3.74, 4.2)	2.03% (1.82, 2.25)
South Asia	44.57 (39.72, 50.2)	6.37 (5.65, 7.19)	30.19 (26.76, 34.11)	4.6 (4.03, 5.22)	209.42 (183.33, 238.85)	12.67 (11.1, 14.5)	108.08 (94.49, 123.38)	6.93 (6.07, 7.94)	2.18% (2.03, 2.33)	1.26% (1.15, 1.37)
Southeast Asia	32.67 (27.65, 39.22)	10.74 (9.11, 12.74)	20.34 (17.04, 24.65)	7.19 (6.06, 8.61)	139.58 (116.51, 168.52)	19.05 (15.97, 22.9)	66.28 (55.07, 80.75)	9.55 (7.99, 11.54)	1.83% (1.76, 1.9)	0.87% (0.79, 0.95)
East Asia	90.56 (73.96, 109.01)	9.12 (7.5, 11)	42.99 (35.35, 52.01)	4.71 (3.91, 5.63)	418.2 (327.55, 520.46)	19.43 (15.21, 24.14)	96.4 (76.55, 119)	4.48 (3.56, 5.52)	2.5% (2.42, 2.58)	−0.43% (−0.54, −0.32)
Oceania	0.55 (0.43, 0.68)	14.57 (11.61, 18.06)	0.32 (0.25, 0.41)	9.69 (7.74, 12.12)	1.57 (1.28, 1.97)	16.34 (13.59, 19.97)	0.96 (0.78, 1.18)	10.9 (9.06, 13.21)	0.21% (0.08, 0.34)	0.37% (0.29, 0.45)
Western Sub‐Saharan Africa	10.2 (8.31, 12.1)	10.6 (8.74, 12.47)	7.58 (6.22, 8.96)	8.44 (6.97, 9.89)	43.48 (32.85, 57.2)	18.49 (14.52, 23.65)	26.25 (20.51, 33.46)	12.4 (10.12, 15.45)	1.89% (1.73, 2.05)	1.32% (1.19, 1.45)
Eastern Sub‐Saharan Africa	9.45 (7.92, 11.39)	11.13 (9.46, 13.34)	7.13 (5.99, 8.62)	9.13 (7.8, 10.96)	34.53 (29.18, 40.79)	16.83 (14.54, 19.56)	21.34 (18.27, 25.02)	11.87 (10.34, 13.8)	1.25% (1.13, 1.38)	0.79% (0.69, 0.88)
Central Sub‐Saharan Africa	2.51 (1.78, 3.39)	9.61 (7.01, 12.66)	1.88 (1.35, 2.52)	7.76 (5.76, 10)	10.1 (7.54, 13.2)	14.96 (11.31, 19.51)	6.36 (4.73, 8.36)	10.61 (8.1, 13.77)	1.45% (1.26, 1.65)	1.04% (0.9, 1.18)
Southern Sub‐Saharan Africa	4.29 (3.61, 4.98)	14.21 (11.8, 16.67)	2.74 (2.28, 3.22)	9.92 (8.14, 11.76)	15.28 (13.7, 16.89)	24.25 (21.92, 26.74)	8.55 (7.74, 9.45)	14.88 (13.56, 16.35)	2.23% (2.01, 2.45)	1.71% (1.47, 1.95)

*Note:* ASIR = age‐standardized incidence rate; ASDR = age‐standardized death rate; DALY = disability‐adjusted life year; EAPC = estimated annual percentage change; UI = uncertainty interval. Values in parentheses are age‐standardized rates per 100,000 population.

At the regional level, the largest increases in ASIR were observed in North Africa and the Middle East, as well as in East Asia, whereas the steepest declines occurred in Northern and Western Europe. These findings underscore substantial heterogeneity across development levels and geographic regions (Supplementary Figure 1). We therefore selected China, the United Kingdom, and India for detailed comparison.

### 3.2 Representative National Transition Profiles across Socioeconomic Settings

To further explore national‐level variations, we selected China, India, and the United Kingdom as representative countries of high‐middle, low‐middle, and high SDI regions, respectively (Figure 2, Table 2). From 1990 to 2021, the ASIR decreased in the United Kingdom from 56.19 to 43.86 per 100,000 (EAPC = −0.77; 95% CI, −0.89 to −0.64), whereas it increased in China from 9.08 to 19.36 per 100,000 (EAPC = 2.50; 95% CI, 2.42–2.58) and in India from 5.92 to 12.15 per 100,000 (EAPC = 2.38; 95% CI, 2.19–2.57). Over the same period, the ASDR declined in China (EAPC = −0.5; 95% CI: −0.62 to −0.39) and the United Kingdom (EAPC = −2.27; 95% CI: −2.36 to −2.19), but increased in India (EAPC = 1.44; 95% CI: 1.29–1.59). In 2021, the estimated numbers of BC deaths were 91,484 in China, 80,818 in India, and 13,914 in the United Kingdom, with corresponding ASDRs of 4.40, 6.52, and 10.62 per 100,000, respectively.

**Figure 2:**
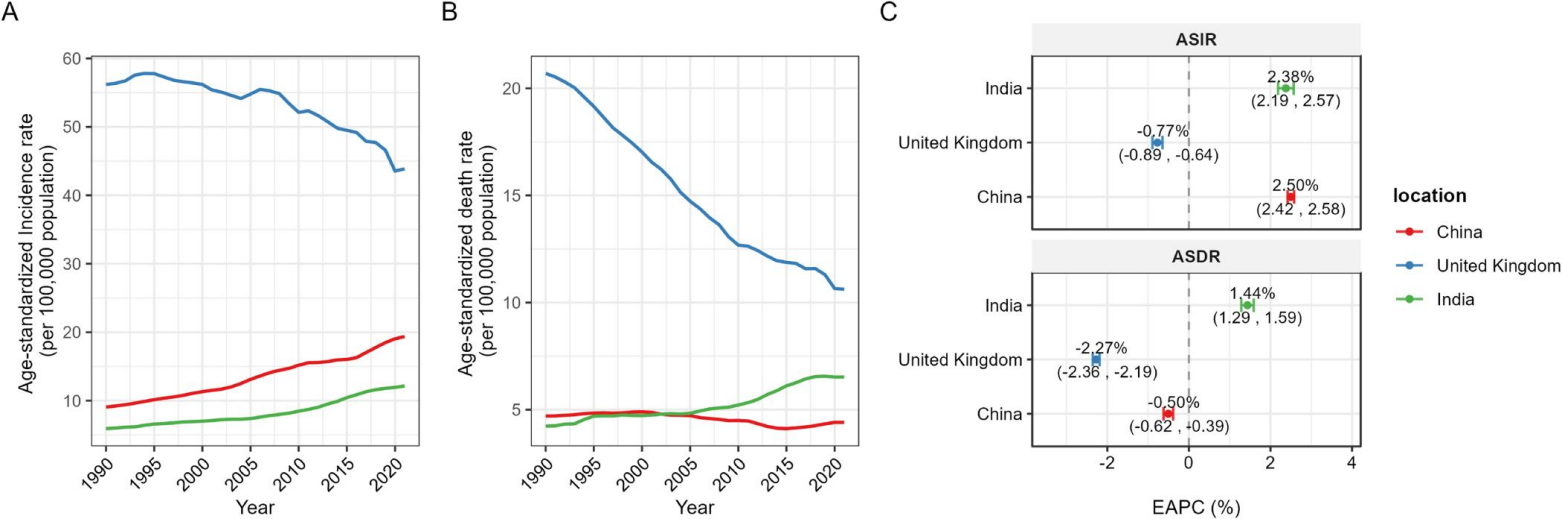
Representative national transition profiles of breast cancer burden across socioeconomic settings (1990–2021). (A) Age‐standardized incidence rate (ASIR) of breast cancer. (B) Age‐standardized death rate (ASDR) of breast cancer. (C) Estimated annual percentage changes (EAPCs) of ASIR and ASDR illustrating distinct transition trajectories across representative countries. Global (grey), China (red), England (blue), and India (green).

**Table 2:** Breast cancer incidence and mortality in China, the United Kingdom, India, and globally: case numbers, age‐standardized rates, and estimated annual percentage changes, 1990–2021.

Location	Deaths_1990	ASMR_1990	Deaths_2021	ASMR_2021	EAPC_Death
Global	355476.52 (335469.79, 373476.11)	9.16 (8.57, 9.61)	674199.41 (623371.55, 720822.55)	7.9 (7.27, 8.44)	−0.59% (−0.64, −0.54)
China	41217.71 (33621.06, 50194.44)	4.7 (3.87, 5.66)	91483.84 (71738.59, 113710.48)	4.4 (3.45, 5.46)	−0.5% (−0.62, −0.39)
United Kingdom	17643.67 (16628.8, 18138.86)	20.69 (19.62, 21.26)	13914.5 (12358.02, 14739.11)	10.62 (9.63, 11.16)	−2.27% (−2.36, −2.19)
India	22510.66 (19225.89, 26349.53)	4.24 (3.57, 5.01)	80817.17 (68415.66, 95686.67)	6.52 (5.52, 7.72)	1.44% (1.29, 1.59)

Location	Incidence_1990	ASIR_1990	Incidence_2021	ASIR_2021	EAPC_Incidence
Global	875657.23 (834228.9, 910528.91)	21.38 (20.27, 22.22)	2121564.32 (1982142.64, 2268722.63)	24.56 (22.93, 26.26)	0.38% (0.33, 0.42)
China	86708.72 (70225.31, 105273.32)	9.08 (7.41, 11.02)	402794.18 (312117.3, 505644.32)	19.36 (15, 24.3)	2.5% (2.42, 2.58)
United Kingdom	44648.02 (42772.11, 45691.67)	56.19 (54.27, 57.43)	49440.22 (45755.97, 51651.32)	43.86 (41.31, 45.51)	−0.77% (−0.89, −0.64)
India	33632.64 (28975.85, 39129.91)	5.92 (5.05, 6.94)	159271.48 (135111.62, 187901.49)	12.15 (10.3, 14.38)	2.38% (2.19, 2.57)

*Note:* ASIR = age‐standardized incidence rate; ASDR = age‐standardized death rate; EAPC = estimated annual percentage change; UI = uncertainty interval. Values in parentheses are age‐standardized rates per 100,000 population.

Absolute age‐standardized incidence and mortality rates differed between GBD 2021 and GLOBOCAN 2022. For example, the corresponding ASIRs were 24.56 versus 46.8 per 100,000 globally, 19.36 versus 33.0 in China, 12.15 versus 26.6 in India, and 43.86 versus 94.0 in the United Kingdom (Supplementary Tables S1 and S2).

To compare mortality relative to incidence across the representative countries, we calculated the MIR as an ecological, rate‐based indicator. Based on the GBD 2021 estimates, India had the highest MIR in 2021 (0.54), followed by the United Kingdom (0.24) and China (0.23) (Supplementary Table S1). The GLOBOCAN 2022 estimates showed a similar broad contrast, with a higher MIR in India (0.52) than in China (0.18) and the United Kingdom (0.15) (Supplementary Table S2). Thus, both data sources indicated higher mortality relative to incidence in India, although their absolute estimates differed and MIR should not be interpreted as a direct measure of patient survival. Additional country‐level estimates and rankings of breast cancer incidence and mortality are presented in Supplementary Table S3. We next analyzed age‐ and sex‐specific distributions of breast cancer burden to characterize demographic differences across countries and socioeconomic settings.

### 3.3 Age‐Structured Transition Patterns of Breast Cancer Burden

Females accounted for approximately 20.25 million of the 20.63 million global breast cancer DALYs in 2021 (98.2%). Among females, the largest age‐specific burden occurred at ages 55–59 years, accounting for 2.83 million of 20.25 million female breast cancer DALYs (14.0%) (Figure 3A). Although total female DALYs increased from 11.04 million in 1990 to 20.25 million in 2021 (+83.5%), the female age‐standardized DALY rate declined from 503.81 to 455.56 per 100,000 population (−9.6%). In males, breast cancer DALYs increased from 0.15 million in 1990 to 0.38 million in 2021 (+162.8%), particularly among individuals aged 55–69 years (Figure 3B). Similar age‐associated patterns were observed for incidence and mortality across sexes (Supplementary Figure 2).

**Figure 3:**
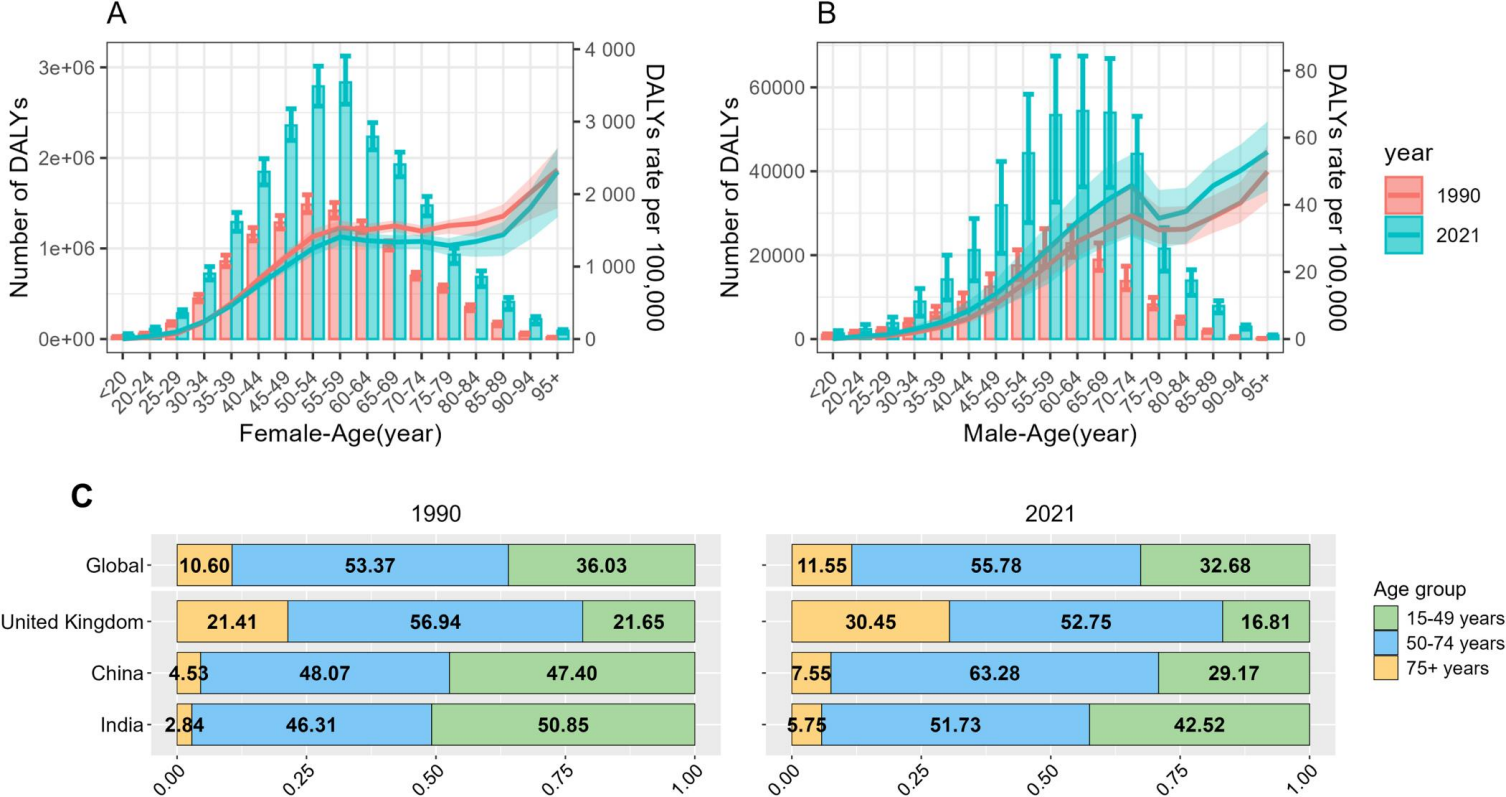
Age‐structured transition patterns of breast cancer DALY burden across representative socioeconomic settings. (A) Female age‐associated transition patterns of breast cancer DALYs in 1990 and 2021. (B) Male age‐associated transition patterns of breast cancer DALYs in 1990 and 2021. (C) Proportion of breast cancer DALYs attributable to three major age groups (15–49, 50–74, and ≥ 75 years) in 1990 and 2021 across global estimates and representative countries (China, India, and the United Kingdom).

Age‐structured burden patterns varied substantially across representative socioeconomic settings, reflecting different stages of epidemiological transition (Figure 3C and Supplementary Table S4). Globally, breast cancer DALYs in 2021 were predominantly concentrated among individuals aged 50–74 years, accounting for 11,509,826 of 20,635,718 DALYs (55.8%), followed by those aged 15–49 years, with 6,743,070 of 20,635,718 DALYs (32.7%), and those aged ≥ 75 years, with 2,382,822 of 20,635,718 DALYs (11.5%). Although individuals aged 50–74 years accounted for the largest proportion of DALYs in all three selected countries, the relative contribution of older age groups differed substantially. Individuals aged ≥ 75 years accounted for 97,408 of 319,901 DALYs (30.4%) in the United Kingdom, compared with 228,788 of 3,029,405 DALYs (7.6%) in China and 158,648 of 2,758,797 DALYs (5.8%) in India. Conversely, individuals aged 15–49 years accounted for 1,172,997 of 2,758,797 DALYs (42.5%) in India, compared with 883,688 of 3,029,405 DALYs (29.2%) in China and 53,761 of 319,901 DALYs (16.8%) in the United Kingdom. The age distribution of deaths showed a similar pattern, with individuals aged ≥ 75 years accounted for 7,513 of 13,914 breast cancer deaths (54.0%) in the United Kingdom, compared with 16,061 of 91,484 deaths (17.6%) in China and 11,559 of 80,818 deaths (14.3%) in India. Detailed age‐specific distributions of DALYs, deaths, and incidence further supported these national profiles (Supplementary Figure 3).

### 3.4 Socioeconomic Transitions in Modifiable Breast Cancer Risk Burden

Globally, the point estimates of the age‐standardized proportions of breast cancer DALYs attributable to high BMI and high FPG increased from 4.22% and 2.76% in 1990 to 4.86% and 3.94% in 2021, respectively. In contrast, the corresponding proportions attributable to high alcohol use and tobacco decreased from 4.41% to 2.82% and from 4.12% to 2.70%, respectively. The proportions attributable to diet high in red meat and low physical activity changed comparatively little, from 12.56% to 11.85% and from 2.06% to 2.01%, respectively. These estimates were interpreted descriptively because the uncertainty intervals for several risk‐attributable proportions were wide.

At the national level, the evolution of risk‐attributable burden differed markedly across the three representative countries (Figure 4A and Supplementary Table S5). In the United Kingdom, age‐standardized DALY rates declined across all six risk factors between 1990 and 2021. The largest reductions were observed for tobacco (−68.5%), diet high in red meat (−50.7%), low physical activity (−49.1%), and alcohol use (−47.4%); high‐BMI‐ and high‐FPG‐attributable rates also decreased by 38.1% and 16.7%, respectively. China showed a mixed pattern: high‐BMI‐ and high‐FPG‐attributable rates increased by 105.0% and 21.7%, respectively, whereas tobacco‐attributable rates declined by 17.8% and red‐meat‐attributable rates remained relatively stable (−2.6%). In India, high‐BMI‐ and high‐FPG‐attributable rates increased by 378.3% and 117.2%, respectively, accompanied by increases in alcohol use (105.3%), diet high in red meat (55.5%), and low physical activity (41.5%); tobacco‐attributable rates changed little (−2.4%).

**Figure 4:**
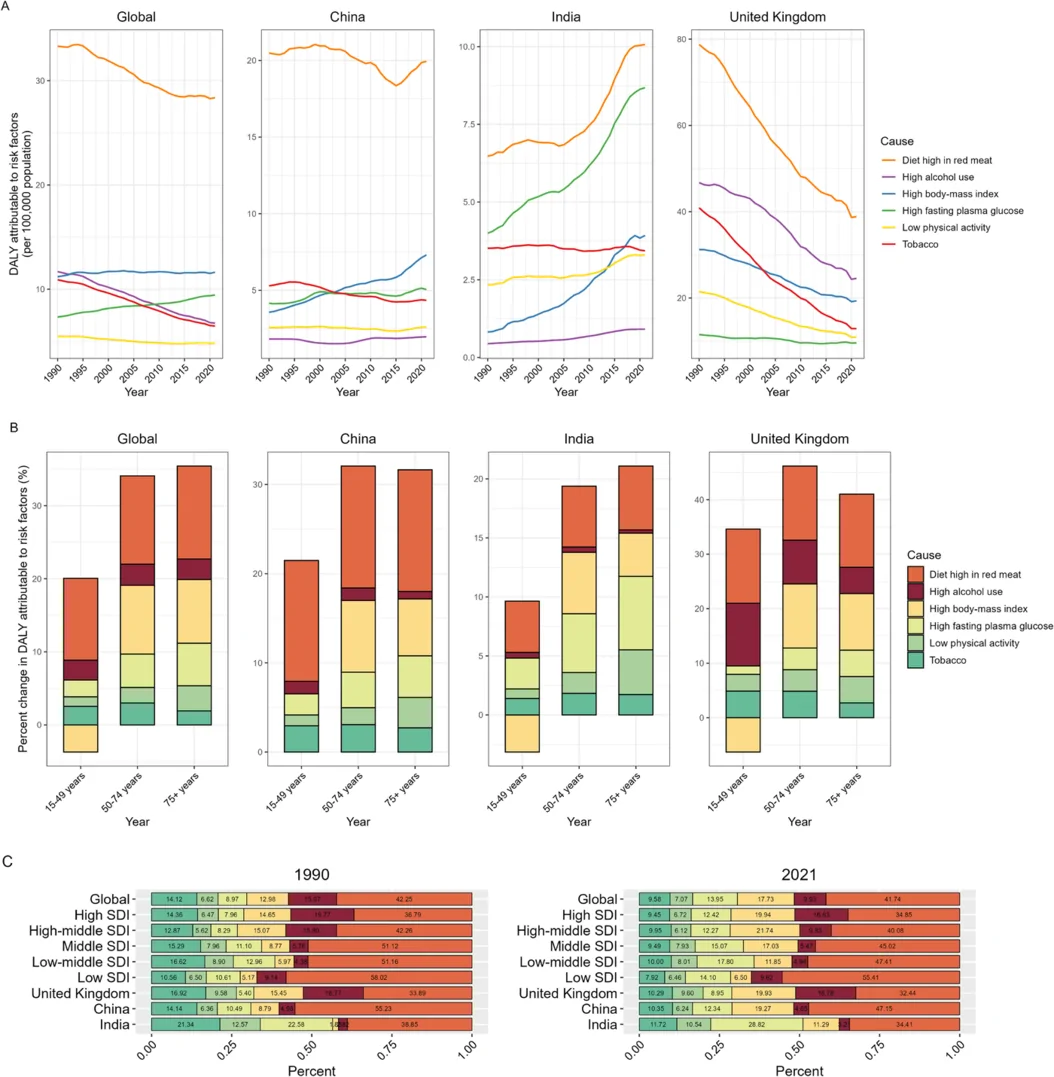
Socioeconomic transitions in behavioral and metabolic risk‐attributable breast cancer burden. (A) Temporal transition patterns of age‐standardized DALY rates attributable to six major modifiable risk factors, including diet high in red meat, alcohol usehigh body‐mass index (BMI), and high fasting plasma glucose (FPG), low physical activity, and tobacco use, from 1990 to 2021. Rates are expressed per 100,000 population. (B) Age‐associated transition patterns in the proportion of breast cancer DALYs attributable to major risk factors in 2021 across three age groups (15–49, 50–74, and ≥ 75 years). (C) Relative composition of DALYs attributable to the six selected risk factors in 1990 and 2021 across global and five Socio‐demographic Index (SDI) quintiles and in China, India, and the United Kingdom.

Age‐specific attributable proportions also differed across countries (Figure 4B; Supplementary Table S6). In the United Kingdom, high alcohol use accounted for 11.51% (95% UI, 7.86%–15.09%) of breast cancer DALYs among individuals aged 15–49 years in 2021, compared with 8.35% (95% UI, 5.79%–11.13%) among those aged 50–74 years and 4.97% (95% UI, 3.03%–7.44%) among those aged ≥ 75 years. Among individuals aged 15–49 years, the corresponding proportions were 1.40% (95% UI, 0.87%–2.08%) in China and 0.49% (95% UI, 0.28%–0.74%) in India. In this age group, the tobacco‐attributable proportion decreased between 1990 and 2021 from 8.21% to 4.81% in the United Kingdom, from 3.14% to 2.94% in China, and from 2.23% to 1.40% in India. In India, the point estimate of the FPG‐attributable proportion increased with age, from 2.60% among individuals aged 15–49 years to 4.97% among those aged 50–74 years and 6.23% among those aged ≥ 75 years in 2021.

Across SDI levels, the dominant attributable risk patterns varied systematically with socioeconomic development. Figure 4C presents the normalized relative composition of the six selected risk‐attributable DALY rates. Diet high in red meat remained the largest component across all SDI categories in 2021, ranging from 34.85% in high‐SDI regions to 55.41% in low‐SDI regions. Meanwhile, the combined relative share of high BMI and high FPG increased from 22.61% to 32.36% in high‐SDI regions, from 23.36% to 34.01% in high‐middle‐SDI regions, and from 18.93% to 29.65% in low‐middle‐SDI regions. These normalized percentages describe the distribution among the six evaluated risk estimates and should not be interpreted as proportions of total breast cancer DALYs because individual risk‐attributable burdens may overlap. Corresponding temporal and regional patterns of breast cancer mortality attributable to these modifiable risk factors are presented in Supplementary Figure 4. Overall, changes in absolute attributable rates and relative risk composition varied across socioeconomic settings rather than following a uniform transition pattern.

### 3.5 Socioeconomic Transition Gradients in Breast Cancer Burden

To further characterize the socioeconomic transition of breast cancer burden, we examined the associations between age‐standardized rates (ASRs) and the Socio‐demographic Index (SDI) across 21 GBD regions. The ASIR was relatively low in regions with SDI < 0.4, increased progressively between SDI 0.4 and 0.7, and rose more steeply at middle‐to‐high SDI levels, reflecting increasing detection capacity, lifestyle westernization, and metabolic risk accumulation during socioeconomic development. However, the slope of increase diminished at the highest SDI levels (> 0.9), resulting in a plateau (Figure 5A), which may indicate saturation of screening coverage and stabilization of incidence in mature healthcare systems. By contrast, the ASDR showed a different pattern (Figure 5B). At low to lower‐middle SDI levels (0–0.4), ASDR remained relatively stable. Between SDI 0.4 and 0.6, ASDR decreased slightly, followed by an increase between 0.6 and 0.75. At higher SDI levels (> 0.75), ASDR declined sharply, reaching the lowest levels in the highest‐SDI countries.

**Figure 5:**
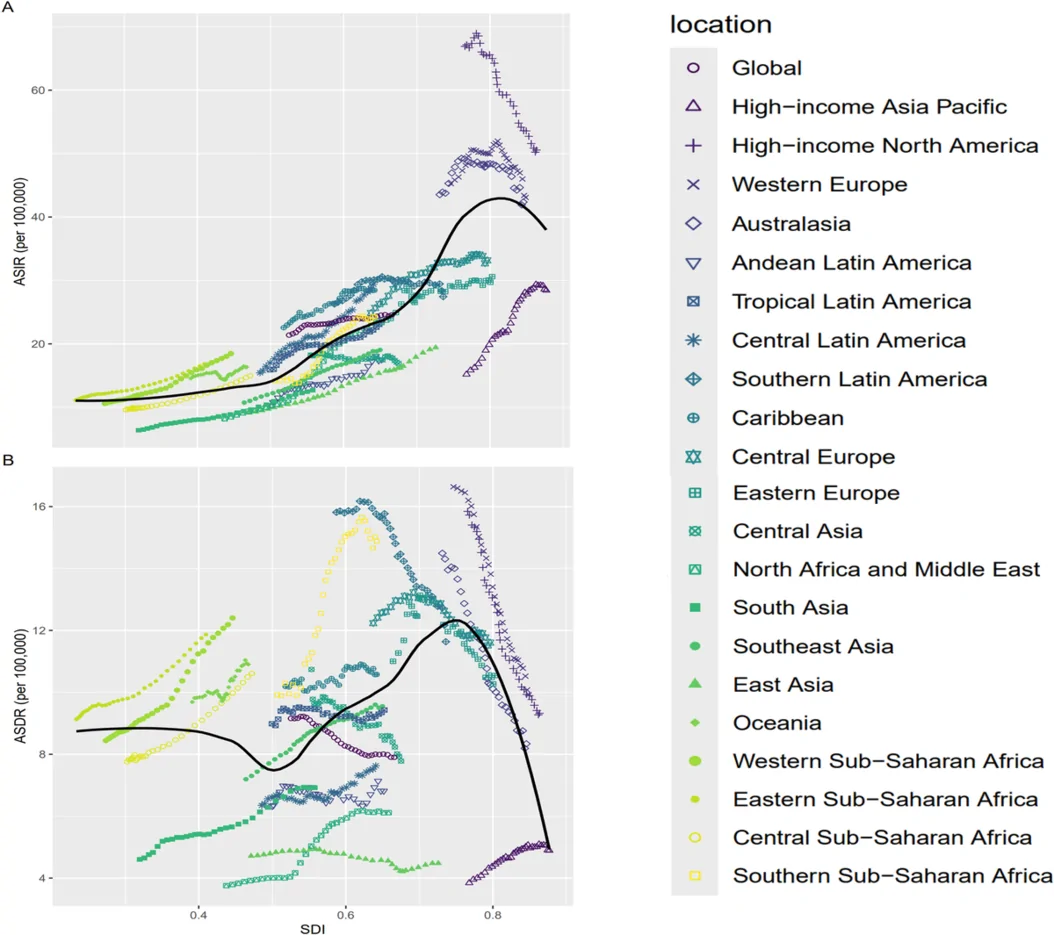
Socioeconomic transition gradients of breast cancer incidence and mortality burden across GBD regions. (A) Association between Socio‐demographic Index (SDI) and age‐standardized incidence rate (ASIR) across 21 GBD regions from 1990 to 2021. (B) Association between SDI and age‐standardized death rate (ASDR) across 21 GBD regions from 1990 to 2021. The black curve represents adaptive Loess regression fitted across all observations, illustrating nonlinear transition trajectories of breast cancer burden during socioeconomic development.

## 4 Discussion

In this comprehensive analysis of the GBD 2021 dataset, we identified substantial heterogeneity in the global burden of breast cancer across time, age, sex, and socioeconomic development. From 1990 to 2021, the global ASIR increased modestly (EAPC, 0.38; 95% CI, 0.33–0.42), whereas the ASDR declined. (EAPC, −0.59; 95% CI, −0.64 to −0.54). This divergence may be consistent with improvements in breast cancer detection and treatment at the population level, although screening participation, stage at diagnosis, and treatment were not directly evaluated in this study [19]. These trends also varied across socioeconomic settings. High‐SDI regions showed decreasing ASIR and ASDR, a pattern also observed in the United Kingdom, where breast cancer mortality declined markedly (ASDR EAPC, −2.27). In high‐middle‐SDI regions, including China, incidence increased while mortality decreased; correspondingly, China showed a declining ASDR (EAPC, −0.50) despite its increasing incidence. In contrast, low‐ and low‐middle‐SDI regions showed increases in both incidence and mortality, a pattern exemplified by India, where the ASDR increased over the study period (EAPC, 1.44). These differences may reflect variation in population structure, risk‐factor exposure, cancer detection and registration, and access to diagnosis and treatment, although these factors were not directly evaluated [20, 21, 22].

Comparison with GLOBOCAN 2022 further demonstrated substantial differences in absolute incidence and mortality estimates between the two data sources. Such discrepancies may arise from differences in reference years, source data, estimation procedures, and age‐standardization methods. GLOBOCAN primarily uses cancer registry and mortality data with country‐specific modeling when direct data are incomplete, whereas GBD integrates multiple data sources within a broader modeling framework to generate internally consistent estimates across locations and years [16, 23]. Therefore, GLOBOCAN was used as an independent contemporary comparison rather than as a quantitative validation of the GBD estimates.

Against this methodological background, the MIR findings should also be interpreted cautiously. India had a higher MIR than China and the United Kingdom, indicating higher mortality relative to incidence at the population level. Cross‐national differences in screening coverage, stage at diagnosis, cancer registration, and access to standardized treatment could potentially contribute to this pattern. However, MIR is an ecological, rate‐based indicator and should not be interpreted as a direct measure of individual survival; differences in MIR may also be influenced by differences in incidence and mortality ascertainment and registration [24].

Age‐ and sex‐specific analyses highlighted further demographic differences. Women accounted for 98.2% of global breast cancer DALYs in 2021, with the largest burden concentrated at ages 50–74 years, while male DALYs increased particularly among individuals aged 55–69 years. This increase may be related to population aging and changes in case detection, although these factors were not directly assessed in the present study [25]. The three representative countries showed distinct age profiles. The United Kingdom exhibited an aging‐associated burden profile, with DALYs concentrated among individuals aged ≥ 75 years, whereas China showed a transitional burden pattern centered in the 50–74‐year population. By contrast, India maintained a disproportionately high burden among younger women aged 15–49 years, exceeding the global average. These differences may arise from a combination of demographic composition, age‐specific disease risk, risk‐factor transitions, and healthcare‐related factors. In higher‐SDI settings, population aging, longer life expectancy, and the strong age‐related increase in breast cancer incidence expand the population at risk at older ages; reductions in premature mortality associated with earlier diagnosis and effective treatment may further contribute to the older distribution of burden [26, 27]. Conversely, India's younger population structure may increase the relative contribution of younger age groups, while deaths occurring at younger ages generate more years of life lost and therefore contribute disproportionately to DALYs [28]. Changes in reproductive and lifestyle‐related exposures, together with differences in early detection, stage at diagnosis, and access to treatment, may further contribute to the burden among younger individuals in lower‐SDI settings, although these potential explanations were not directly assessed [29, 30].

Risk‐attributable burden evolved differently across socioeconomic settings rather than following a uniform transition toward metabolic predominance. A diet high in red meat remained the leading contributor to breast cancer DALYs globally, although its proportional contribution experienced a pronounced decline in high‐SDI settings. This trend may reflect the effectiveness of public dietary guidelines and health promotion campaigns that have reduced red meat consumption in many high‐SDI countries since the 1990s [31]. In China, the attributable DALY rate remained comparatively stable during the early study period, subsequently declined until the mid‐2010s, and then partially rebounded toward 2021. This pattern may be related to dietary composition and the relatively high consumption of pork and other red meat products reported in previous studies [32]. India had the lowest absolute rate among the three countries but showed a sustained increase over the study period. Efforts to reduce red meat–related risk may therefore be insufficient in regions with limited health promotion resources and in populations where red meat consumption is deeply embedded in dietary culture [33].

Alcohol‐ and tobacco‐attributable DALYs declined in high‐SDI regions, consistent with the success of public health regulations. Align with public health policy implementation reported in the packaging, taxation and price regulation of both alcohol and tobacco [34, 35, 36, 37, 38]. However, the effects of individual policies were not directly evaluated in the present study. Age‐specific attributable fractions also revealed residual heterogeneity. In the United Kingdom, high alcohol use accounted for a larger proportion of breast cancer DALYs among individuals aged 15–49 years (11.51%) than among those aged 50–74 years (8.35%) or ≥ 75 years (4.97%). The corresponding proportion in the younger age group was also higher than that observed in China (1.40%) and India (0.49%), suggesting that alcohol‐related burden may remain particularly relevant among younger individuals in the United Kingdom. In contrast, the tobacco‐attributable proportion declined in all three countries, from 8.21% to 4.81% in the United Kingdom, from 3.14% to 2.94% in China, and from 2.23% to 1.40% in India.

Metabolic risk patterns likewise varied across the three countries. In the United Kingdom, the age‐standardized DALY rates attributable to high BMI and high FPG decreased between 1990 and 2021. Nevertheless, their relative contributions among the six risk factors examined increased, indicating a compositional shift toward metabolic risks within an overall declining attributable burden, as several behavioral risk‐attributable rates decreased more rapidly. In contrast, the increasing DALY rates attributable to high BMI and high FPG in rapidly developing settings indicate a growing contribution of metabolic risks to BC burden. Although the high‐BMI‐attributable DALY burden among individuals aged 15–49 years decreased globally, China did not show a comparable decline. This discrepancy is consistent with national surveys showing escalating obesity rates among Chinese adolescents and young adults [39]. Such trends underscore the urgency of implementing early‐life interventions that address unhealthy diet to curb the long‐term impact of excess weight on cancer risk [40, 41]. In India, FPG emerged as a particularly important metabolic risk factor in the GBD attribution framework, especially among women aged 50–74 years. This aligns with the well‐documented epidemic of type 2 diabetes in South Asia, driven by genetic predisposition, high rates of central obesity, and dietary patterns rich in refined carbohydrate [42]. However, the present ecological analysis did not directly assess individual glycemic status or its association with BC outcomes. These findings highlight the need for integrated cancer and diabetes prevention strategies in India, including improved glycemic control and healthier dietary patterns.

From a policy perspective, different stages of breast cancer epidemiological transition require distinct prevention and control strategies across socioeconomic settings. High‐SDI countries should prioritize optimizing screening intervals and long‐term survivorship care, as the burden shifts to older populations. In rapidly developing regions such as China, interventions targeting obesity, unhealthy dietary patterns, and metabolic dysfunction are urgently needed. In lower‐SDI settings such as India, strengthening early detection, expanding treatment access, and addressing the dual challenges of early‐onset BC and metabolic risk are critical priorities [43].

Several limitations should be noted. First, GBD 2021 estimates are derived from heterogeneous data sources and modeling approaches, and data quality remains uneven across regions. Consequently, estimates from data‐sparse settings may be more dependent on modeling assumptions, potentially affecting the precision. Second, only selected behavioral and metabolic risk factors were included; other relevant determinants, such as genetic predisposition, molecular subtypes, and treatment accessibility, were not captured. The reported attributable burden should therefore not be interpreted as representing the complete risk‐attributable or preventable burden of breast cancer. Third, MIR was calculated using population‐level incidence and mortality rates from the same calendar year. It may be affected by differences in case ascertainment, registry completeness, population structure, and the timing of incidence and death events and should not be interpreted as a direct measure of individual survival or treatment effectiveness. Finally, the estimates reflect historical trends through 2021, and continued surveillance is necessary to monitor future epidemiological transitions in BC burden.

## 5 Conclusion

Globally, breast cancer incidence continues to rise despite declining mortality, yet substantial disparities persist across socioeconomic settings. High‐SDI regions increasingly exhibit aging‐associated survivorship patterns, whereas rapidly developing regions face growing metabolic risk burden and lower‐SDI regions continue to experience disproportionate early‐onset disease and healthcare inequality. These findings highlight distinct epidemiological transition patterns of breast cancer and underscore the importance of region‐specific strategies integrating prevention, metabolic risk control, early detection, and equitable healthcare access.

## Author Contributions

**Zhangyue Sun:** conceptualization, methodology, formal analysis, writing – original draft. **Xiaolu Tan:** methodology, software, formal analysis, writing – review and editing. **Qianrui Xu:** writing – review and editing, software. **Hong Liang:** writing – review and editing, supervision. **Jie Chen:** supervision, writing – original draft, writing – review and editing.

## Funding

The authors have nothing to report.

## Ethics Statement

This study involved no human or animal participants. All data were obtained from the publicly available Global Burden of Disease 2021 database and were fully de‐identified prior to analysis. Ethical approval and informed consent were not required.

## Conflicts of Interest

The authors declare no conflicts of interest.

## Transparency Statement

All authors have read and approved the final version of the manuscript. Jie Chen had full access to all of the data in this study and takes complete responsibility for the integrity of the data and the accuracy of the data analysis. Jie Chen affirms that this manuscript is an honest, accurate, and transparent account of the study being reported; that no important aspects of the study have been omitted; and that any discrepancies from the study as planned have been explained.

## Supporting information


Supporting File 1

Supporting File 2

## Data Availability

The data that support the findings of this study are publicly available from the Global Burden of Disease (GBD) 2021 study through the Global Health Data Exchange (GHDx) at https://ghdx.healthdata.org/gbd-results-tool. Additional cancer burden estimates were obtained from the GLOBOCAN 2022 database, available at https://gco.iarc.who.int/. All data used in this study are publicly accessible and do not require special permission.
