# Supplementary material for: Epidemiological Transitions in Breast Cancer Burden across Socioeconomic Settings, 1990–2021: An Ecological Time‐Trend Study of China, the United Kingdom, and India
Source: Health Sci Rep. 2026 Nov 10;9(11):e73479. doi: 10.1002/hsr2.73479 (PMC13749273; doi:10.1002/hsr2.73479)
Supplement: Supplementary file 1 [file HSR2-9-e73479-s002.pdf]

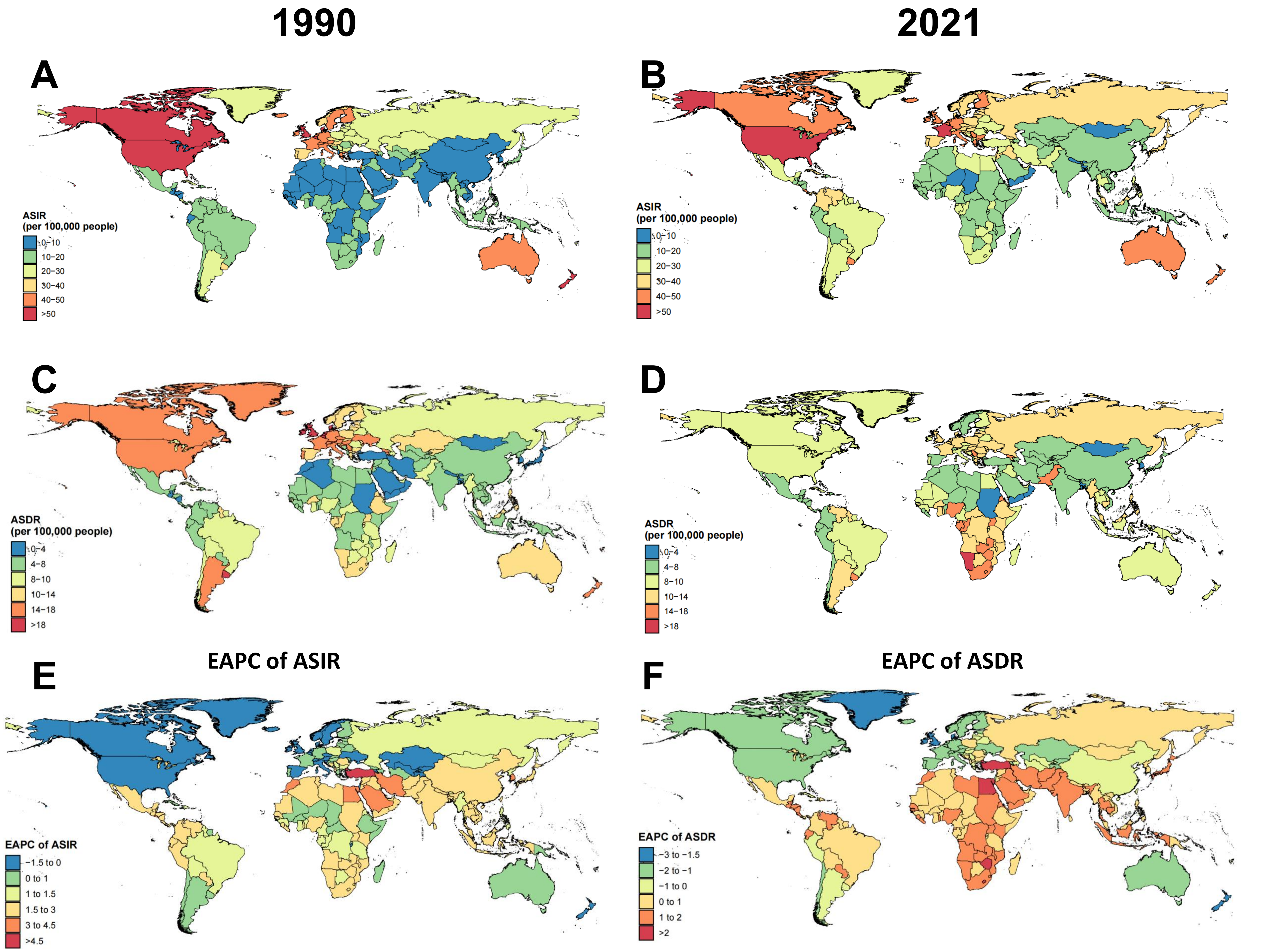

Supplementary Figure1. Global burden and trend of BC in 204 countries or territories (1990-2021). **(A)** The ASIR per 100,000 people in 1990; **(B)** The ASIR per 100,000 people in 20021; **(C)** The ASDR per 100,000 people in 1990; **(D)** The ASDR per 100,000 people in 20021. **(E)** The EAPC for BC incidence during 1990 to 2021; **(F)** The EAPC for BC death during 1990 to 2021.

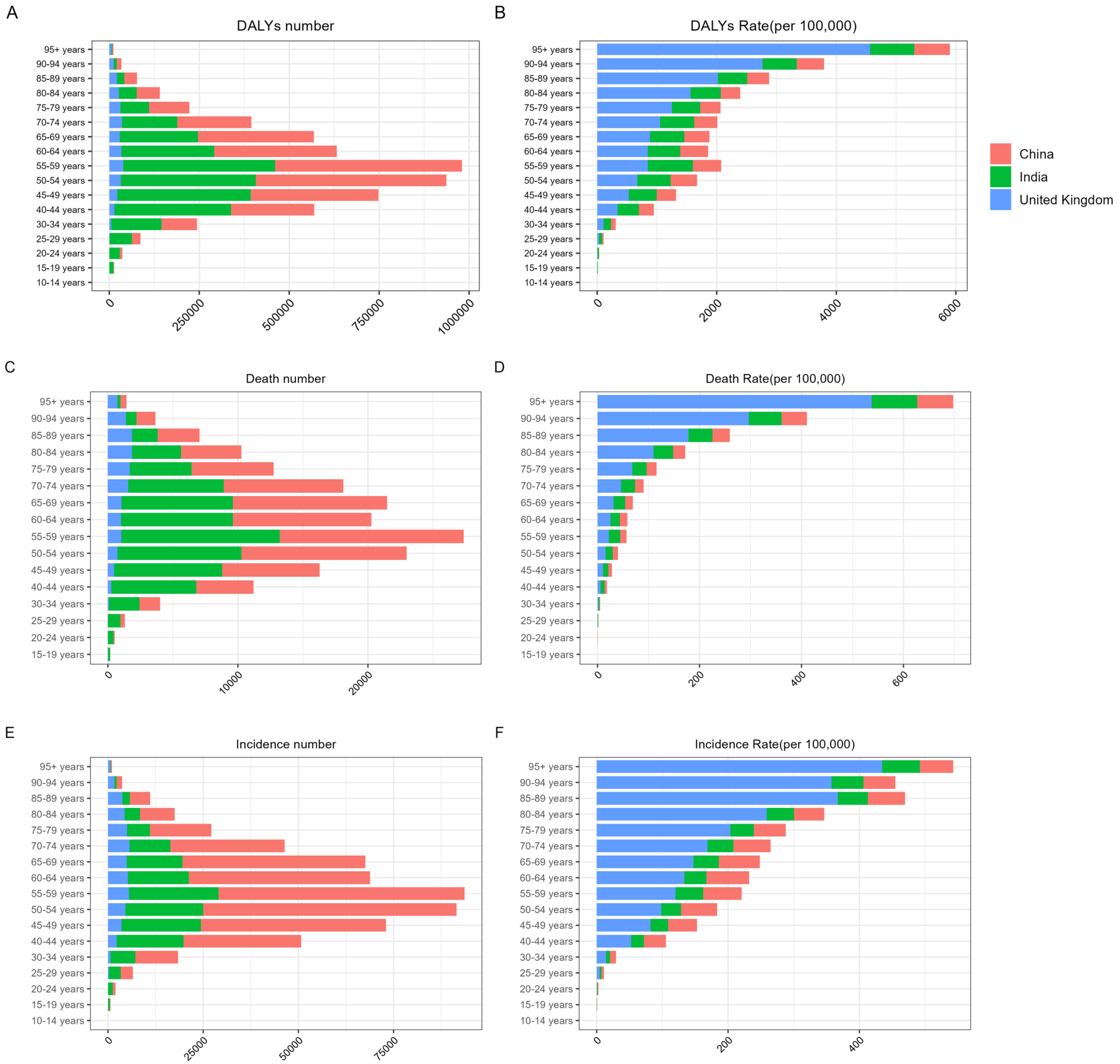

Supplementary Figure2. Age-specific number and rate (per 100,000 population) of BC in 2021 for China, India, and the United Kingdom.
(A-B) DALYs; (C-D) Deaths; (E-F) Incidence

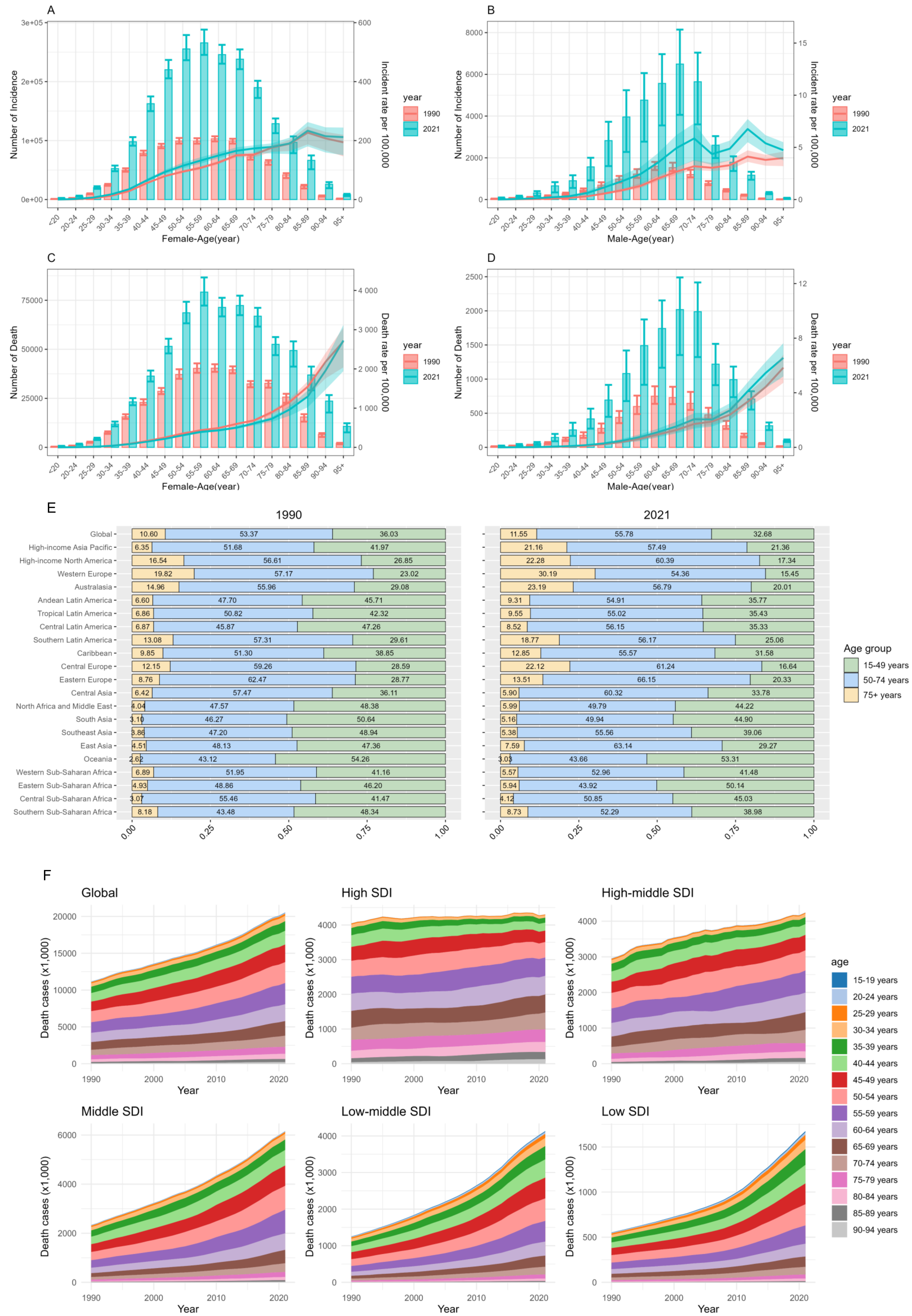

Supplementary Figure3. Age- and sex-specific global breast cancer burden.
(A–B) Incidence and **(C–D)** deaths of breast cancer in females and males in 1990 and 2021 by age group, shown as numbers (bars) with 95% UIs and rates per 100,000 population (lines with shaded areas).
(E) Proportion of BC deaths by age group (15–49, 50–74, ≥75 years) across global and 21 GBD regions in 1990 and 2021 (F) Global and SDI-level trends in breast cancer deaths by age group from 1990 to 2021.

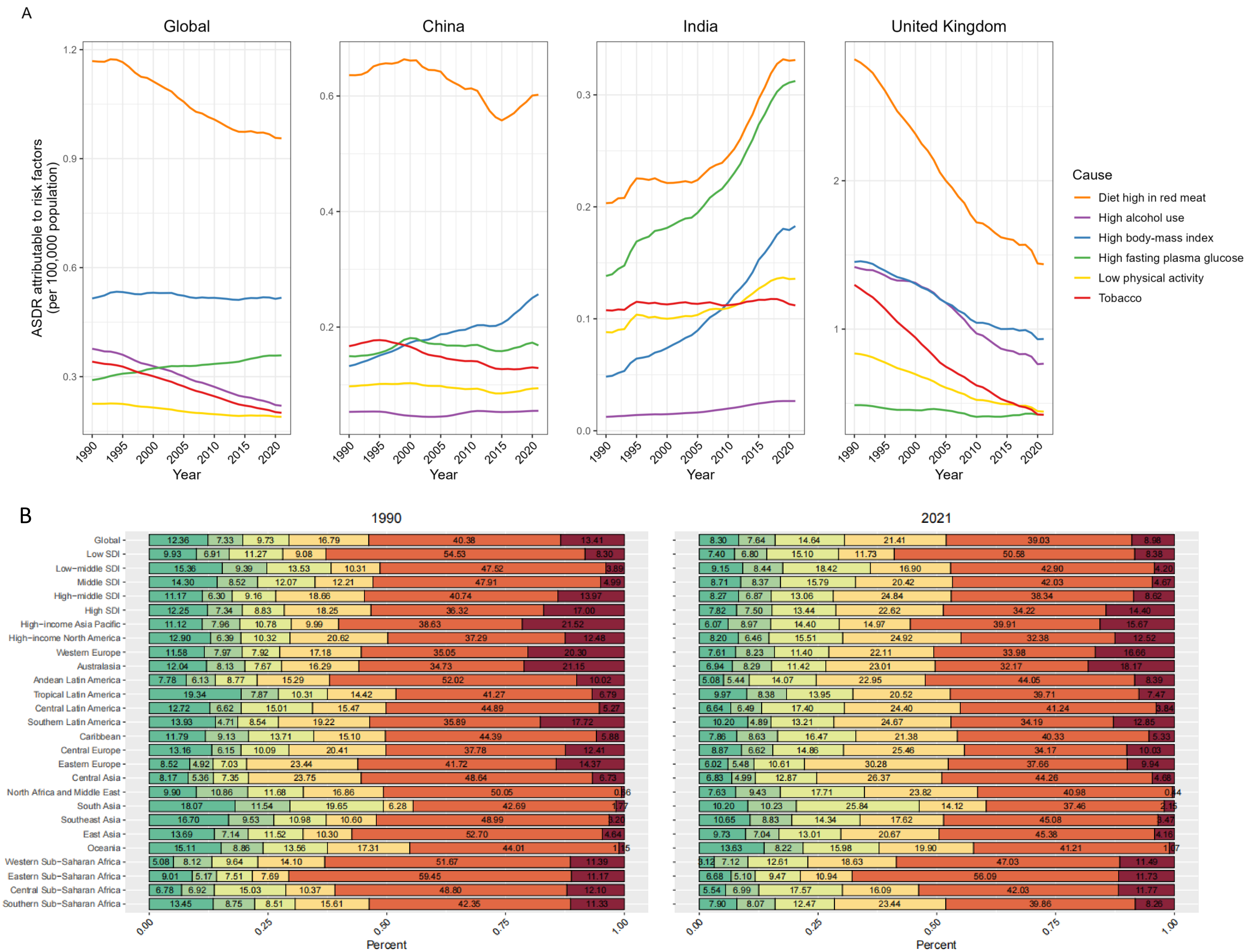

Supplementary Figure4. Breast cancer mortality attributable to modifiable risk factors.

(A) Temporal trends in age-standardized death rates (ASDRs) attributable to six major risk factors (diet high in red meat, alcohol use, high body-mass index, high fasting plasma glucose, low physical activity, and tobacco) globally and in three representative countries (China, India, and the United Kingdom) from 1990 to 2021.

(B) Relative composition of breast cancer mortality attributable to the six selected risk factors globally, across five SDI regions, and across 21 GBD regions in 1990 and 2021.
