# Supplementary material for: Epidemiological Transitions in Breast Cancer Burden across Socioeconomic Settings, 1990–2021: An Ecological Time‐Trend Study of China, the United Kingdom, and India
Source: Health Sci Rep. 2026 Nov 10;9(11):e73479. doi: 10.1002/hsr2.73479 (PMC13749273; doi:10.1002/hsr2.73479)
Supplement: Supplementary file 2 [file HSR2-9-e73479-s001.docx]

Supplementary Online Content

Global and national trends in breast cancer burden and risk factors, 1990–2021: socioeconomic and age-specific heterogeneity in China, the UK, and India

Supplemental data include 5 tables.

Supplementary Table S1. Comparison of age-standardized incidence rates, death rates, and mortality-to-incidence ratios of breast cancer in GBD 2021

Supplementary Table S2. Current breast cancer burden and mortality-to-incidence ratios (MIRs) in representative countries based on GLOBOCAN 2022 estimates

Supplementary Table S3. Country-level rankings and burden estimates of breast cancer incidence and mortality in 204 countries and territories, 1990 and 2021.

Supplementary Table S4. Age distribution of breast cancer DALYs and deaths by SDI quintile and representative countries in 2021.

Supplementary Table S5. Age-standardized DALY rates of breast cancer attributable to selected modifiable risk factors globally and in representative countries, 1990 and 2021.

Supplementary Table S6. Age-specific proportions of breast cancer deaths and disability-adjusted life years attributable to selected risk factors in China, the United Kingdom, and India, 1990 and 2021

This supplementary material has been provided by the authors to give readers additional information about their work.

Supplementary Table S1. Comparison of age-standardized incidence rates, death rates, and mortality-to-incidence ratios of breast cancer in GBD 2021

| Location | ASIR 2021 | ASDR 2021 | MIR 2021 |
| --- | --- | --- | --- |
| Global | 24.56 | 7.90 | 0.32 |
| China | 19.36 | 4.40 | 0.23 |
| United Kingdom | 43.86 | 10.62 | 0.24 |
| India | 12.15 | 6.52 | 0.54 |

Footnote: MIR was calculated as the ratio of the age-standardized death rate to the age-standardized incidence rate. It is an ecological, rate-based indicator of mortality relative to incidence and should not be interpreted as a direct measure of patient survival. Incident cases and deaths occurring in the same calendar year do not necessarily represent the same patient cohort.

Supplementary Table S2. Current breast cancer burden and mortality-to-incidence ratios (MIRs) in representative countries based on GLOBOCAN 2022 estimates.

| Location | Incidence (2022) | Mortality (2022) | ASIR | ASDR | MIR2022 |
| --- | --- | --- | --- | --- | --- |
| Global | 2295833 | 665683 | 46.8 | 12.7 | 0.27 |
| China | 357161 | 74986 | 33 | 6.1 | 0.18 |
| India | 192020 | 98337 | 26.6 | 13.7 | 0.52 |
| United Kingdom | 58756 | 12122 | 94 | 14 | 0.15 |

Footnote: Data were obtained from GLOBOCAN 2022 and are presented as point estimates, as uncertainty intervals were not available in the downloaded GCO summary outputs.MIR was calculated as the ratio of the age-standardized Death rate (ASDR) to the age-standardized incidence rate (ASIR). A higher MIR may indirectly reflect poorer survival

| Supplementary Table S3. Country-level rankings and burden estimates of breast cancer incidence and mortality in 204 countries and territories, 1990 and 2021. | | | | | | | | | | | | | | | | |
| --- | --- | --- | --- | --- | --- | --- | --- | --- | --- | --- | --- | --- | --- | --- | --- | --- |
| Location | Incident cases 1990 | Incident cases 2021 | % change in incident cases | Incident cases rank 2021 | ASIR 1990 | ASIR 2021 | % change in ASIR | ASIR rank 2021 | Deaths 1990 | Deaths 2021 | % change in deaths | Deaths rank 2021 | ASDR 1990 | ASDR 2021 | % change in ASDR | ASDR rank 2021 |
| China | 86708.72 | 402794.18 | 364.54 | 1 | 9.08 | 19.36 | 113.34 | 130 | 41217.71 | 91483.84 | 121.95 | 1 | 4.7 | 4.4 | -6.27 | 193 |
| United States of America | 204920.21 | 272387.51 | 32.92 | 2 | 68.32 | 51.76 | -24.24 | 6 | 49187.61 | 53472.76 | 8.71 | 3 | 15.94 | 9.41 | -40.95 | 113 |
| India | 33632.64 | 159271.48 | 373.56 | 3 | 5.92 | 12.15 | 105.2 | 184 | 22510.66 | 80817.17 | 259.02 | 2 | 4.24 | 6.52 | 53.99 | 172 |
| Japan | 28197.83 | 78401.01 | 178.04 | 4 | 16.98 | 31.93 | 88.03 | 65 | 6605.09 | 16825.97 | 154.74 | 9 | 3.95 | 5.42 | 37.01 | 185 |
| Russian Federation | 39422.71 | 76078.63 | 92.98 | 5 | 22.22 | 33.56 | 51.01 | 55 | 17353.86 | 25303.93 | 45.81 | 5 | 9.83 | 10.83 | 10.15 | 82 |
| Germany | 49694.68 | 73076.59 | 47.05 | 6 | 42.08 | 44.96 | 6.83 | 14 | 20718.64 | 20860.86 | 0.69 | 7 | 16.84 | 10.76 | -36.11 | 84 |
| Brazil | 16011.13 | 61092.71 | 281.56 | 7 | 16 | 23.79 | 48.72 | 107 | 8381.64 | 23850.7 | 184.56 | 6 | 9.05 | 9.43 | 4.2 | 112 |
| France | 32633.97 | 59205.37 | 81.42 | 8 | 43.51 | 52.55 | 20.79 | 5 | 12971.14 | 15358.11 | 18.4 | 11 | 16.2 | 10.62 | -34.48 | 89 |
| Italy | 38620.24 | 51530.18 | 33.43 | 9 | 48.63 | 44.23 | -9.06 | 17 | 13040 | 14640.86 | 12.28 | 12 | 15.56 | 9.89 | -36.42 | 101 |
| Indonesia | 12228.86 | 49532.23 | 305.04 | 10 | 10.14 | 17.39 | 71.53 | 145 | 8000.83 | 25997.73 | 224.94 | 4 | 7.16 | 9.94 | 38.79 | 100 |
| United Kingdom | 44648.02 | 49440.22 | 10.73 | 11 | 56.19 | 43.86 | -21.93 | 18 | 17643.67 | 13914.5 | -21.14 | 13 | 20.69 | 10.62 | -48.69 | 88 |
| Mexico | 7427.9 | 36561.66 | 392.22 | 12 | 14.8 | 26.95 | 82.15 | 93 | 2830.54 | 9498.09 | 235.56 | 15 | 6.2 | 7.29 | 17.54 | 157 |
| Pakistan | 7881.68 | 34748.76 | 340.88 | 13 | 12.41 | 22.64 | 82.33 | 118 | 5634.07 | 20310.27 | 260.49 | 8 | 9.32 | 14.67 | 57.29 | 35 |
| Turkey | 2440.83 | 28899.68 | 1084.01 | 14 | 6.1 | 29.92 | 390.05 | 78 | 1117.95 | 6370.77 | 469.86 | 23 | 2.96 | 6.81 | 130.08 | 167 |
| Spain | 17607.39 | 26779.74 | 52.09 | 15 | 36.03 | 32.79 | -8.99 | 60 | 6748.12 | 7213.61 | 6.9 | 20 | 13.23 | 7.29 | -44.87 | 156 |
| Nigeria | 5525.66 | 26098.66 | 372.32 | 16 | 11.73 | 23.95 | 104.16 | 106 | 4197.26 | 15668.45 | 273.3 | 10 | 9.5 | 15.92 | 67.49 | 25 |
| Canada | 17032.53 | 25610.49 | 50.36 | 17 | 53.65 | 40.76 | -24.03 | 30 | 4822.11 | 6110.66 | 26.72 | 24 | 15.1 | 8.57 | -43.23 | 136 |
| Iran (Islamic Republic of) | 3200.96 | 24764.09 | 673.65 | 18 | 9.9 | 26.46 | 167.24 | 94 | 926.61 | 4065.29 | 338.73 | 31 | 3.23 | 4.75 | 47.16 | 192 |
| Thailand | 4594.03 | 24635.62 | 436.25 | 19 | 10.46 | 24.43 | 133.48 | 102 | 2371.32 | 9093.5 | 283.48 | 16 | 5.89 | 8.62 | 46.39 | 133 |
| Philippines | 5321.76 | 21561.45 | 305.16 | 20 | 14.94 | 22.65 | 51.63 | 117 | 3215.85 | 11381.64 | 253.92 | 14 | 10.12 | 12.86 | 27.03 | 50 |
| Poland | 9631.96 | 21058.68 | 118.63 | 21 | 22.58 | 32.69 | 44.79 | 61 | 5060.08 | 8615.04 | 70.25 | 17 | 11.97 | 12.09 | 0.99 | 56 |
| Egypt | 3006.22 | 20680.03 | 587.91 | 22 | 8.5 | 27.3 | 221.31 | 91 | 1402.99 | 6000.37 | 327.68 | 25 | 4.36 | 8.77 | 101.07 | 128 |
| Colombia | 3829.67 | 19094.81 | 398.6 | 23 | 18.71 | 34.75 | 85.71 | 51 | 1423.56 | 4234 | 197.42 | 29 | 7.6 | 7.66 | 0.73 | 148 |
| Australia | 7756.03 | 16366.47 | 111.02 | 24 | 40.84 | 41.54 | 1.72 | 26 | 2613.48 | 3606.11 | 37.98 | 35 | 13.83 | 8.12 | -41.32 | 144 |
| Republic of Korea | 2589.74 | 16186.35 | 525.02 | 25 | 6.81 | 19.55 | 186.95 | 127 | 1020.31 | 2879.01 | 182.17 | 39 | 2.96 | 3.3 | 11.32 | 202 |
| Viet Nam | 3221.11 | 15969.79 | 395.79 | 26 | 7.63 | 14.55 | 90.66 | 170 | 1997.73 | 6400.61 | 220.39 | 22 | 4.91 | 6.23 | 26.96 | 174 |
| Argentina | 9039.82 | 15948.8 | 76.43 | 27 | 28.18 | 29.49 | 4.65 | 82 | 5436.7 | 7373.69 | 35.63 | 19 | 17.22 | 13.25 | -23.04 | 45 |
| Ukraine | 19088.5 | 15687.53 | -17.82 | 28 | 28.15 | 21.89 | -22.24 | 121 | 9738.11 | 7628.63 | -21.66 | 18 | 14.08 | 10.18 | -27.72 | 95 |
| Netherlands | 9286.06 | 13450.15 | 44.84 | 29 | 49.37 | 45.62 | -7.61 | 13 | 3728.61 | 3776.98 | 1.3 | 34 | 19.14 | 10.96 | -42.73 | 76 |
| Bangladesh | 2482.66 | 13210.87 | 432.13 | 30 | 4.02 | 8.36 | 108.19 | 199 | 1638.56 | 5764.77 | 251.82 | 26 | 2.76 | 3.75 | 35.6 | 201 |
| South Africa | 3454.39 | 12094.18 | 250.11 | 31 | 14.87 | 24.29 | 63.39 | 103 | 2166.76 | 6556.01 | 202.57 | 21 | 10.12 | 14.36 | 41.84 | 38 |
| Venezuela (Bolivarian Republic of) | 2164.41 | 12010.33 | 454.9 | 32 | 18.8 | 38.57 | 105.17 | 35 | 788.56 | 3240.75 | 310.97 | 37 | 7.47 | 10.69 | 43.09 | 86 |
| Taiwan (Province of China) | 2136.25 | 10867.26 | 408.71 | 33 | 11.65 | 28.61 | 145.55 | 84 | 781.54 | 2844.4 | 263.95 | 40 | 4.61 | 7.04 | 52.72 | 161 |
| Romania | 5009.29 | 9728.08 | 94.2 | 34 | 18.35 | 29.93 | 63.08 | 77 | 2656.59 | 4135.61 | 55.67 | 30 | 9.77 | 11.49 | 17.56 | 64 |
| Myanmar | 3435.44 | 9610.09 | 179.73 | 35 | 12.35 | 17.62 | 42.68 | 140 | 2400.56 | 5317.65 | 121.52 | 27 | 9.08 | 10.21 | 12.49 | 93 |
| Iraq | 1256.63 | 9554.25 | 660.31 | 36 | 13.35 | 31.2 | 133.79 | 69 | 536.6 | 2309.45 | 330.39 | 45 | 6.08 | 8.43 | 38.74 | 139 |
| Malaysia | 1859.53 | 9434.87 | 407.38 | 37 | 16.52 | 30.28 | 83.29 | 76 | 1074.3 | 3899.61 | 262.99 | 33 | 10.27 | 13.23 | 28.83 | 46 |
| Greece | 6055.95 | 8771.19 | 44.84 | 38 | 43.33 | 44.28 | 2.19 | 16 | 1998.3 | 2907.47 | 45.5 | 38 | 13.86 | 11.75 | -15.23 | 58 |
| Belgium | 7681.9 | 8666.36 | 12.82 | 39 | 55.28 | 43.41 | -21.47 | 20 | 2936.67 | 2585.97 | -11.94 | 42 | 20 | 10.83 | -45.84 | 81 |
| Ethiopia | 2619.7 | 7869.31 | 200.39 | 40 | 11.75 | 14.95 | 27.24 | 165 | 2075.58 | 4875.35 | 134.89 | 28 | 10.26 | 10.55 | 2.81 | 90 |
| Portugal | 4421.93 | 7310.21 | 65.32 | 41 | 35.33 | 38.34 | 8.54 | 36 | 1827.71 | 2103.44 | 15.09 | 48 | 14.39 | 8.75 | -39.16 | 129 |
| Peru | 1467.41 | 6360.71 | 333.46 | 42 | 10.87 | 18.04 | 65.91 | 136 | 830.15 | 2119.51 | 155.32 | 47 | 6.48 | 6.14 | -5.24 | 177 |
| Democratic Republic of the Congo | 1680.74 | 6205.87 | 269.23 | 43 | 9.23 | 14.02 | 51.92 | 175 | 1251.78 | 3995.03 | 219.15 | 32 | 7.38 | 10.12 | 37.2 | 97 |
| Saudi Arabia | 491.48 | 6190.14 | 1159.5 | 44 | 6.29 | 18.43 | 193.1 | 134 | 207.95 | 1223.25 | 488.24 | 74 | 2.87 | 4.05 | 41 | 197 |
| Hungary | 4458.25 | 6160.56 | 38.18 | 45 | 32.24 | 36.06 | 11.84 | 44 | 2152.56 | 2222.8 | 3.26 | 46 | 15.42 | 11.62 | -24.6 | 61 |
| Algeria | 1029.72 | 6065.4 | 489.03 | 46 | 7.71 | 14.88 | 93.16 | 166 | 452.29 | 1541.06 | 240.72 | 60 | 3.81 | 4.22 | 10.7 | 196 |
| Sweden | 5150.61 | 5996.81 | 16.43 | 47 | 40.45 | 32.88 | -18.7 | 59 | 1669.15 | 1613.97 | -3.31 | 59 | 11.98 | 7.31 | -38.98 | 155 |
| Czechia | 4226.64 | 5949.54 | 40.76 | 48 | 31.86 | 31.53 | -1.03 | 68 | 1948.48 | 1982.79 | 1.76 | 51 | 14.46 | 9.27 | -35.89 | 117 |
| Serbia | 3272.3 | 5924.72 | 81.06 | 49 | 29.32 | 40.4 | 37.82 | 32 | 1644.14 | 2342.12 | 42.45 | 44 | 15.75 | 14.63 | -7.11 | 36 |
| Cuba | 2699.61 | 5885.31 | 118.01 | 50 | 26.21 | 32.13 | 22.61 | 64 | 998.06 | 1845.4 | 84.9 | 53 | 9.87 | 9.48 | -4 | 110 |
| Morocco | 921.38 | 5619.46 | 509.9 | 51 | 5.68 | 14.81 | 160.58 | 167 | 453.51 | 1768.15 | 289.88 | 56 | 2.96 | 4.86 | 63.95 | 188 |
| United Republic of Tanzania | 1454.45 | 5378.9 | 269.82 | 52 | 12.2 | 17.62 | 44.4 | 141 | 1051.39 | 3300.48 | 213.92 | 36 | 9.7 | 12.23 | 26.1 | 54 |
| Austria | 4607.49 | 5296.47 | 14.95 | 53 | 43.14 | 33.88 | -21.47 | 53 | 1871.22 | 1730.15 | -7.54 | 58 | 16.4 | 9.2 | -43.95 | 118 |
| Switzerland | 3819.9 | 5294.88 | 38.61 | 54 | 40.05 | 32.97 | -17.68 | 58 | 1426.61 | 1495.35 | 4.82 | 62 | 14.07 | 7.9 | -43.88 | 146 |
| Bulgaria | 3359.95 | 5071.09 | 50.93 | 55 | 28.89 | 41.37 | 43.2 | 28 | 1312.58 | 1758.5 | 33.97 | 57 | 11.42 | 13.19 | 15.47 | 48 |
| Chile | 1758.52 | 4987.21 | 183.6 | 56 | 16.73 | 20.33 | 21.56 | 125 | 974.11 | 1776.37 | 82.36 | 55 | 9.76 | 7 | -28.35 | 163 |
| Kenya | 846.17 | 4823.77 | 470.07 | 57 | 8.91 | 17.4 | 95.26 | 143 | 576.64 | 2789.18 | 383.7 | 41 | 6.57 | 11.48 | 74.75 | 66 |
| Democratic People's Republic of Korea | 1718.32 | 4536.36 | 164 | 58 | 9.22 | 13.3 | 44.31 | 179 | 992.16 | 2072.53 | 108.89 | 49 | 5.72 | 6.18 | 8.09 | 175 |
| Sri Lanka | 1105.89 | 4474.85 | 304.64 | 59 | 8.77 | 16.64 | 89.79 | 149 | 629.4 | 1779.31 | 182.7 | 54 | 5.48 | 6.69 | 22.08 | 170 |
| Belarus | 2941.95 | 4445.58 | 51.11 | 60 | 23.64 | 29.74 | 25.81 | 80 | 1247.79 | 1362.95 | 9.23 | 68 | 9.87 | 8.73 | -11.59 | 130 |
| Finland | 2700.89 | 4276.42 | 58.33 | 61 | 40.41 | 41.86 | 3.59 | 24 | 907.59 | 1036.85 | 14.24 | 80 | 13.17 | 8.27 | -37.2 | 143 |
| Uzbekistan | 1637.11 | 4243.62 | 159.21 | 62 | 13.26 | 13.18 | -0.64 | 180 | 896.12 | 1846.72 | 106.08 | 52 | 7.54 | 6.16 | -18.22 | 176 |
| Israel | 1890.74 | 4171.62 | 120.63 | 63 | 40.98 | 36.71 | -10.43 | 41 | 768.67 | 1270 | 65.22 | 72 | 16.72 | 10.17 | -39.17 | 96 |
| Uganda | 884.91 | 4063.85 | 359.24 | 64 | 12.61 | 22.7 | 79.94 | 115 | 660.69 | 2485.35 | 276.17 | 43 | 10.12 | 15.5 | 53.16 | 26 |
| Denmark | 3746.8 | 3860.95 | 3.05 | 65 | 51.9 | 37.59 | -27.56 | 38 | 1734.62 | 1324.79 | -23.63 | 70 | 22.45 | 10.89 | -51.5 | 78 |
| Ghana | 923.37 | 3584.5 | 288.2 | 66 | 12.18 | 17.57 | 44.2 | 142 | 626.28 | 2037.48 | 225.33 | 50 | 9.03 | 11.26 | 24.68 | 68 |
| Kazakhstan | 3077.95 | 3539.7 | 15 | 67 | 22.73 | 18.15 | -20.14 | 135 | 1638.36 | 1380.2 | -15.76 | 66 | 12.6 | 7.56 | -40.02 | 150 |
| Tunisia | 636.54 | 3487.14 | 447.83 | 68 | 11.63 | 25.1 | 115.83 | 99 | 244.97 | 782.53 | 219.44 | 91 | 4.86 | 5.85 | 20.36 | 180 |
| New Zealand | 2145.27 | 3483.02 | 62.36 | 69 | 57.16 | 46.62 | -18.44 | 10 | 673.29 | 761.71 | 13.13 | 93 | 17.85 | 9.48 | -46.85 | 109 |
| Syrian Arab Republic | 627.26 | 3371.25 | 437.46 | 70 | 9.71 | 22.84 | 135.28 | 114 | 255.94 | 772.63 | 201.88 | 92 | 4.3 | 5.46 | 26.87 | 183 |
| Ireland | 1860.7 | 3126.35 | 68.02 | 71 | 49.13 | 43.34 | -11.79 | 21 | 704.6 | 722.08 | 2.48 | 97 | 18.34 | 9.28 | -49.42 | 116 |
| Lebanon | 555.89 | 3123.36 | 461.86 | 72 | 24.18 | 52.72 | 118 | 4 | 221.9 | 674.64 | 204.03 | 98 | 10.3 | 11.11 | 7.88 | 74 |
| Jordan | 296.49 | 3030.9 | 922.27 | 73 | 17.05 | 32.6 | 91.19 | 62 | 110.92 | 621.9 | 460.66 | 100 | 7.09 | 7.53 | 6.25 | 151 |
| Slovakia | 1472.48 | 2989.99 | 103.06 | 74 | 25.22 | 33.24 | 31.83 | 57 | 701.74 | 1043.03 | 48.63 | 79 | 11.99 | 11.21 | -6.53 | 70 |
| Sudan | 522.61 | 2853.4 | 445.99 | 75 | 4.75 | 10.65 | 124.01 | 191 | 279.51 | 945.35 | 238.21 | 83 | 2.73 | 3.9 | 42.59 | 199 |
| Croatia | 2004.52 | 2798.59 | 39.61 | 76 | 33.32 | 36.09 | 8.3 | 43 | 831.25 | 957.52 | 15.19 | 81 | 14.37 | 10.8 | -24.89 | 83 |
| Ecuador | 468.85 | 2765.76 | 489.91 | 77 | 7.8 | 16.37 | 109.84 | 154 | 267.36 | 1107.89 | 314.38 | 77 | 4.76 | 6.77 | 42.17 | 168 |
| Norway | 2045.7 | 2699.52 | 31.96 | 78 | 34.4 | 30.9 | -10.17 | 70 | 855.45 | 735.69 | -14 | 95 | 13.24 | 7.22 | -45.5 | 159 |
| Cambodia | 520.5 | 2672.96 | 413.53 | 79 | 9.78 | 18.98 | 93.95 | 131 | 381.62 | 1511.43 | 296.06 | 61 | 7.66 | 11.53 | 50.66 | 62 |
| Singapore | 581.34 | 2452.39 | 321.85 | 80 | 21.42 | 28.35 | 32.37 | 85 | 196.37 | 483.5 | 146.21 | 113 | 7.95 | 5.6 | -29.64 | 182 |
| Costa Rica | 435.07 | 2402.06 | 452.1 | 81 | 22.43 | 43.77 | 95.16 | 19 | 118.44 | 492.78 | 316.06 | 111 | 6.5 | 8.93 | 37.51 | 124 |
| Zambia | 391.86 | 2390.53 | 510.04 | 82 | 11.47 | 25.1 | 118.73 | 100 | 286.73 | 1396.13 | 386.92 | 65 | 9.24 | 16.97 | 83.67 | 17 |
| C么te d'Ivoire | 518.34 | 2319 | 347.39 | 83 | 10.74 | 16.68 | 55.21 | 148 | 367.31 | 1374.49 | 274.2 | 67 | 8.48 | 11.22 | 32.29 | 69 |
| Angola | 399.15 | 2291.13 | 474 | 84 | 8.33 | 15.2 | 82.46 | 162 | 301.05 | 1401.52 | 365.54 | 64 | 6.85 | 10.52 | 53.65 | 91 |
| Cameroon | 518.03 | 2235.4 | 331.52 | 85 | 10.2 | 14.41 | 41.31 | 172 | 371.36 | 1332.87 | 258.92 | 69 | 7.87 | 9.76 | 24.06 | 103 |
| Mozambique | 636.82 | 2143.32 | 236.56 | 86 | 9.76 | 16.52 | 69.32 | 150 | 491.61 | 1446.76 | 194.29 | 63 | 8.35 | 12.94 | 54.89 | 49 |
| Nepal | 556.42 | 2141.31 | 284.84 | 87 | 4.93 | 8.37 | 69.73 | 198 | 399.18 | 1165.33 | 191.93 | 76 | 3.78 | 4.83 | 27.85 | 189 |
| Puerto Rico | 1016.09 | 2106.58 | 107.32 | 88 | 28.54 | 36.95 | 29.45 | 39 | 365.5 | 591.85 | 61.93 | 101 | 10.44 | 8.82 | -15.46 | 127 |
| Azerbaijan | 907.64 | 2063.93 | 127.39 | 89 | 16.83 | 17.38 | 3.27 | 146 | 507.79 | 867.87 | 70.91 | 89 | 9.85 | 7.78 | -21.05 | 147 |
| Madagascar | 642.11 | 2048.06 | 218.96 | 90 | 11.07 | 13.9 | 25.58 | 176 | 473.03 | 1263.94 | 167.2 | 73 | 8.72 | 9.85 | 13.03 | 102 |
| Afghanistan | 523.01 | 2043.43 | 290.7 | 91 | 7.25 | 14.29 | 97.16 | 174 | 335.89 | 941.4 | 180.27 | 84 | 4.71 | 7.27 | 54.43 | 158 |
| Uruguay | 1353.79 | 2036.06 | 50.4 | 92 | 36.99 | 41.12 | 11.17 | 29 | 755.37 | 933.25 | 23.55 | 85 | 20.14 | 16.85 | -16.34 | 18 |
| Zimbabwe | 527.3 | 2004.08 | 280.06 | 93 | 12 | 23.98 | 99.75 | 105 | 358.93 | 1285.74 | 258.22 | 71 | 8.99 | 17.01 | 89.33 | 16 |
| Georgia | 2033.6 | 1963.39 | -3.45 | 94 | 33.21 | 35.95 | 8.27 | 45 | 959.7 | 948.32 | -1.19 | 82 | 15.61 | 16.4 | 5.04 | 22 |
| Haiti | 566.72 | 1803.28 | 218.2 | 95 | 15.49 | 20.54 | 32.59 | 124 | 394.11 | 1079.69 | 173.96 | 78 | 11.56 | 13.41 | 15.98 | 44 |
| Burkina Faso | 626.58 | 1794.5 | 186.39 | 96 | 13.39 | 16.48 | 23.07 | 151 | 459.36 | 1167.7 | 154.2 | 75 | 10.65 | 12.08 | 13.43 | 57 |
| Bolivia (Plurinational State of) | 422.93 | 1772.57 | 319.12 | 97 | 11.73 | 17.86 | 52.21 | 138 | 283.57 | 890.28 | 213.95 | 87 | 8.37 | 9.53 | 13.86 | 105 |
| Yemen | 240.95 | 1722.16 | 614.73 | 98 | 4.09 | 9.2 | 124.98 | 195 | 130.67 | 664.4 | 408.48 | 99 | 2.44 | 3.94 | 61.51 | 198 |
| Dominican Republic | 448.82 | 1714.85 | 282.08 | 99 | 10.74 | 16.36 | 52.3 | 155 | 239.63 | 755.46 | 215.26 | 94 | 6.32 | 7.41 | 17.26 | 153 |
| Guatemala | 228.24 | 1642.4 | 619.61 | 100 | 5.57 | 13.51 | 142.32 | 177 | 114.35 | 548.53 | 379.71 | 108 | 3.19 | 4.8 | 50.63 | 191 |
| El Salvador | 308.63 | 1636.7 | 430.3 | 101 | 9.51 | 26.98 | 183.7 | 92 | 132.87 | 422.23 | 217.76 | 121 | 4.26 | 6.84 | 60.63 | 166 |
| Panama | 291.97 | 1627.68 | 457.49 | 102 | 17.83 | 36.87 | 106.78 | 40 | 91.07 | 332.16 | 264.75 | 132 | 5.91 | 7.46 | 26.37 | 152 |
| Republic of Moldova | 1145.71 | 1586.3 | 38.46 | 103 | 25.23 | 27.68 | 9.69 | 88 | 544.42 | 590.55 | 8.47 | 102 | 12.3 | 10.05 | -18.27 | 98 |
| Libya | 211.23 | 1563.44 | 640.17 | 104 | 9.64 | 23.3 | 141.62 | 111 | 83.51 | 396.9 | 375.27 | 126 | 4.1 | 6.53 | 59.18 | 171 |
| Lithuania | 1186.02 | 1486.71 | 25.35 | 105 | 27.26 | 30.51 | 11.93 | 75 | 471.2 | 567.1 | 20.35 | 106 | 10.67 | 10.18 | -4.51 | 94 |
| United Arab Emirates | 113.29 | 1475.84 | 1202.67 | 106 | 17.05 | 29.08 | 70.58 | 83 | 44.1 | 335.25 | 660.27 | 130 | 8.31 | 8.9 | 7.05 | 125 |
| Bosnia and Herzegovina | 680.85 | 1468.67 | 115.71 | 107 | 15.24 | 25.73 | 68.78 | 98 | 319.47 | 577.02 | 80.61 | 105 | 7.59 | 9.49 | 25.14 | 108 |
| Rwanda | 514.81 | 1424.4 | 176.68 | 108 | 15.91 | 19.45 | 22.27 | 129 | 400.36 | 888.29 | 121.87 | 88 | 13.31 | 13.78 | 3.57 | 41 |
| Malawi | 397.84 | 1421.22 | 257.23 | 109 | 9.07 | 16 | 76.43 | 157 | 299.39 | 902.09 | 201.31 | 86 | 7.32 | 11.51 | 57.12 | 63 |
| Paraguay | 296.93 | 1402.68 | 372.4 | 110 | 12.1 | 22.47 | 85.66 | 119 | 152.89 | 565.83 | 270.1 | 107 | 6.59 | 9.51 | 44.36 | 106 |
| Mali | 429.54 | 1310.44 | 205.08 | 111 | 9.44 | 12.33 | 30.64 | 183 | 318.6 | 827.75 | 159.81 | 90 | 7.41 | 8.44 | 13.85 | 138 |
| Honduras | 193.16 | 1268.98 | 556.96 | 112 | 8.09 | 17.73 | 119 | 139 | 91.15 | 473.43 | 419.42 | 114 | 4.12 | 7.14 | 73.16 | 160 |
| Jamaica | 407.23 | 1243.64 | 205.39 | 113 | 23.89 | 40.4 | 69.14 | 31 | 189.66 | 463.26 | 144.26 | 116 | 10.9 | 14.73 | 35.11 | 33 |
| Slovenia | 780.79 | 1233.63 | 58 | 114 | 32.37 | 31.66 | -2.2 | 66 | 319.93 | 431.06 | 34.74 | 120 | 13.1 | 9.14 | -30.21 | 119 |
| Kuwait | 143.74 | 1204.42 | 737.94 | 115 | 15.9 | 26.35 | 65.64 | 95 | 34.16 | 165.31 | 383.96 | 148 | 4.79 | 4.39 | -8.38 | 194 |
| Palestine | 209.11 | 1202.27 | 474.95 | 116 | 21.69 | 39.19 | 80.7 | 34 | 87.78 | 300.74 | 242.61 | 135 | 9.79 | 11.19 | 14.22 | 71 |
| Senegal | 278.99 | 1189.64 | 326.41 | 117 | 7.74 | 13.42 | 73.4 | 178 | 201.81 | 729.84 | 261.65 | 96 | 5.97 | 8.99 | 50.62 | 122 |
| Armenia | 943.51 | 1170.45 | 24.05 | 118 | 32.05 | 27.56 | -14.01 | 89 | 454.09 | 503.05 | 10.78 | 110 | 15.99 | 11.65 | -27.16 | 60 |
| North Macedonia | 490.12 | 1119.09 | 128.33 | 119 | 24.46 | 34.97 | 42.96 | 49 | 234.71 | 417.68 | 77.96 | 123 | 12.16 | 13.93 | 14.58 | 40 |
| Latvia | 930.76 | 1074.68 | 15.46 | 120 | 26.97 | 30.54 | 13.25 | 74 | 417.76 | 422.07 | 1.03 | 122 | 11.96 | 10.84 | -9.32 | 80 |
| Cyprus | 261.75 | 957.14 | 265.66 | 121 | 33.25 | 49.42 | 48.63 | 8 | 101.19 | 212.43 | 109.94 | 143 | 13.82 | 11.39 | -17.61 | 67 |
| Nicaragua | 148.11 | 955.1 | 544.88 | 122 | 7.84 | 17.26 | 120.15 | 147 | 56.68 | 248.89 | 339.09 | 139 | 3.31 | 4.82 | 45.74 | 190 |
| Papua New Guinea | 281.45 | 954.88 | 239.28 | 123 | 11.44 | 13.05 | 14.13 | 181 | 170.84 | 583.81 | 241.73 | 103 | 7.54 | 8.66 | 14.75 | 132 |
| Lao People's Democratic Republic | 213.96 | 823.6 | 284.92 | 124 | 9.1 | 14.62 | 60.58 | 169 | 164.15 | 488.35 | 197.49 | 112 | 7.39 | 9.43 | 27.59 | 111 |
| Kyrgyzstan | 539.32 | 811.38 | 50.45 | 125 | 17.49 | 14.34 | -18.02 | 173 | 305.98 | 335.2 | 9.55 | 131 | 10.18 | 6.42 | -36.95 | 173 |
| Congo | 184.4 | 807.38 | 337.85 | 126 | 15.61 | 23.07 | 47.8 | 112 | 138.28 | 470.7 | 240.38 | 115 | 12.54 | 15.22 | 21.39 | 29 |
| Somalia | 284.9 | 803.56 | 182.05 | 127 | 9.2 | 10.5 | 14.16 | 193 | 216.37 | 577.13 | 166.73 | 104 | 7.76 | 8.58 | 10.47 | 134 |
| Guinea | 284.74 | 799.75 | 180.87 | 128 | 8.05 | 12.09 | 50.08 | 186 | 219.18 | 529.8 | 141.71 | 109 | 6.4 | 8.58 | 34.11 | 135 |
| Tajikistan | 368.98 | 766.22 | 107.66 | 129 | 12.4 | 10.16 | -18.04 | 194 | 218.28 | 376.14 | 72.32 | 128 | 7.62 | 5.44 | -28.59 | 184 |
| Estonia | 585.56 | 734.92 | 25.51 | 130 | 29.62 | 30.86 | 4.16 | 72 | 253.12 | 247.92 | -2.05 | 140 | 12.65 | 9.09 | -28.14 | 120 |
| Turkmenistan | 277.08 | 694.94 | 150.81 | 131 | 12.87 | 14.54 | 13.02 | 171 | 156.85 | 303.92 | 93.77 | 134 | 7.7 | 6.75 | -12.31 | 169 |
| Togo | 141.42 | 694.72 | 391.27 | 132 | 9.28 | 14.77 | 59.04 | 168 | 97.69 | 413.81 | 323.61 | 124 | 7.03 | 10.02 | 42.61 | 99 |
| Trinidad and Tobago | 239.98 | 663.71 | 176.57 | 133 | 27.6 | 35.45 | 28.47 | 46 | 119.39 | 257.47 | 115.65 | 137 | 14.69 | 13.68 | -6.89 | 42 |
| Niger | 175 | 661.26 | 277.86 | 134 | 5.29 | 6.88 | 30.18 | 203 | 131.21 | 443.43 | 237.94 | 117 | 4.35 | 5.08 | 16.79 | 187 |
| Eritrea | 183.79 | 658.47 | 258.28 | 135 | 12.97 | 19.5 | 50.42 | 128 | 137.95 | 437.22 | 216.95 | 119 | 10.86 | 14.95 | 37.75 | 31 |
| Burundi | 302.69 | 650.21 | 114.81 | 136 | 11.83 | 10.92 | -7.7 | 189 | 237.28 | 438.76 | 84.91 | 118 | 9.85 | 8.39 | -14.85 | 142 |
| Benin | 166.33 | 644.9 | 287.72 | 137 | 7.67 | 10.51 | 37.07 | 192 | 125.16 | 406.19 | 224.53 | 125 | 6.05 | 7.37 | 21.87 | 154 |
| Albania | 200.63 | 620.03 | 209.04 | 138 | 8.77 | 16.26 | 85.44 | 156 | 105.38 | 229.52 | 117.8 | 141 | 5.04 | 5.69 | 12.9 | 181 |
| Qatar | 42.54 | 603.84 | 1319.6 | 139 | 21.15 | 42.59 | 101.36 | 22 | 12.34 | 82.45 | 568.16 | 161 | 8.83 | 8.44 | -4.44 | 137 |
| Chad | 184.09 | 584.58 | 217.55 | 140 | 6.16 | 8.25 | 33.92 | 200 | 141.89 | 394.82 | 178.25 | 127 | 4.95 | 6.13 | 23.88 | 178 |
| South Sudan | 237.57 | 578.79 | 143.63 | 141 | 8.63 | 12.06 | 39.84 | 187 | 180.47 | 371.6 | 105.91 | 129 | 7.01 | 8.71 | 24.3 | 131 |
| Bahrain | 61.06 | 531.03 | 769.68 | 142 | 25.3 | 44.81 | 77.08 | 15 | 22.29 | 98.81 | 343.36 | 154 | 11.48 | 10.64 | -7.32 | 87 |
| Sierra Leone | 143.69 | 504.9 | 251.37 | 143 | 6.53 | 11.03 | 69.03 | 188 | 108.04 | 315.26 | 191.79 | 133 | 5.17 | 7.62 | 47.43 | 149 |
| Mauritius | 89.16 | 503.9 | 465.18 | 144 | 10.91 | 28.29 | 159.36 | 86 | 48.61 | 224.61 | 362.1 | 142 | 6.49 | 12.47 | 92.15 | 53 |
| Namibia | 96.22 | 477.64 | 396.42 | 145 | 13.55 | 29.91 | 120.66 | 79 | 67.65 | 267.2 | 294.99 | 136 | 10.27 | 18.31 | 78.26 | 12 |
| Montenegro | 226.33 | 418.47 | 84.89 | 146 | 35.29 | 45.79 | 29.76 | 12 | 79.05 | 136.04 | 72.1 | 151 | 12.62 | 14.8 | 17.3 | 32 |
| Mauritania | 103.72 | 382.98 | 269.23 | 147 | 9.86 | 15.69 | 59.08 | 158 | 78.67 | 209.81 | 166.68 | 144 | 7.85 | 9.31 | 18.7 | 115 |
| Luxembourg | 244.08 | 377.47 | 54.65 | 148 | 47.34 | 37.8 | -20.16 | 37 | 99.68 | 104.12 | 4.46 | 152 | 18.94 | 9.51 | -49.8 | 107 |
| Central African Republic | 138.33 | 346.75 | 150.67 | 149 | 10.42 | 12.63 | 21.22 | 182 | 108.96 | 254.25 | 133.35 | 138 | 8.88 | 10.34 | 16.48 | 92 |
| Liberia | 86.7 | 342.68 | 295.23 | 150 | 7.07 | 12.13 | 71.68 | 185 | 66.47 | 196.81 | 196.07 | 145 | 5.71 | 7.99 | 40.03 | 145 |
| Malta | 193.91 | 331.16 | 70.78 | 151 | 45.48 | 41.48 | -8.79 | 27 | 80.95 | 99.37 | 22.76 | 153 | 19.3 | 10.72 | -44.45 | 85 |
| Botswana | 77.19 | 313.18 | 305.75 | 152 | 12.64 | 18.56 | 46.8 | 133 | 53.56 | 183.93 | 243.44 | 146 | 9.7 | 12.48 | 28.61 | 52 |
| Gabon | 88.94 | 282.57 | 217.72 | 153 | 15.17 | 23.49 | 54.9 | 110 | 66.07 | 157.97 | 139.1 | 149 | 11.76 | 14.71 | 25.04 | 34 |
| Barbados | 106.18 | 259.64 | 144.54 | 154 | 40.91 | 54.72 | 33.76 | 2 | 49.1 | 96.21 | 95.94 | 155 | 18.06 | 19.41 | 7.47 | 8 |
| Fiji | 120.09 | 250.54 | 108.62 | 155 | 25.71 | 30.73 | 19.52 | 73 | 66.68 | 156.11 | 134.13 | 150 | 16.37 | 21.06 | 28.65 | 2 |
| Oman | 35.38 | 243.77 | 588.99 | 156 | 4.21 | 9.09 | 115.99 | 196 | 14.23 | 47.59 | 234.56 | 169 | 1.94 | 2.16 | 11.19 | 204 |
| Lesotho | 88.91 | 242.65 | 172.9 | 157 | 10.19 | 21.21 | 108.1 | 122 | 67.31 | 169.45 | 151.73 | 147 | 8.16 | 16.13 | 97.68 | 23 |
| Bahamas | 73.42 | 230.76 | 214.32 | 158 | 41.44 | 52.82 | 27.45 | 3 | 32.19 | 85.48 | 165.57 | 159 | 19.47 | 20.66 | 6.14 | 5 |
| Iceland | 127.16 | 216.03 | 69.89 | 159 | 47.41 | 41.87 | -11.68 | 23 | 40.16 | 51.92 | 29.3 | 168 | 14.4 | 8.95 | -37.82 | 123 |
| Guyana | 74.42 | 184.86 | 148.4 | 160 | 17.22 | 26.2 | 52.15 | 96 | 44.17 | 90.87 | 105.74 | 157 | 10.92 | 13.59 | 24.4 | 43 |
| Mongolia | 48.91 | 181.86 | 271.83 | 161 | 4.18 | 6.3 | 50.75 | 204 | 32.65 | 84.84 | 159.85 | 160 | 2.92 | 3.28 | 12.66 | 203 |
| Equatorial Guinea | 21.79 | 162.97 | 647.85 | 162 | 9.93 | 24.27 | 144.35 | 104 | 17.01 | 81.99 | 381.92 | 163 | 8.26 | 14.23 | 72.31 | 39 |
| Eswatini | 41.04 | 146.43 | 256.82 | 163 | 12.78 | 22.95 | 79.6 | 113 | 28.74 | 90.47 | 214.76 | 158 | 10 | 16.08 | 60.73 | 24 |
| Guinea-Bissau | 46.07 | 143.92 | 212.4 | 164 | 9.85 | 15.05 | 52.71 | 164 | 35.07 | 93.91 | 167.8 | 156 | 8.04 | 11.13 | 38.47 | 73 |
| Djibouti | 21.44 | 138.82 | 547.48 | 165 | 12.49 | 17.4 | 39.38 | 144 | 14.94 | 82.29 | 450.72 | 162 | 9.91 | 12.2 | 23.08 | 55 |
| Suriname | 42.79 | 130.48 | 204.97 | 166 | 15.51 | 20.17 | 29.99 | 126 | 23.68 | 61.72 | 160.62 | 165 | 8.99 | 9.73 | 8.31 | 104 |
| Brunei Darussalam | 23.22 | 111.49 | 380.26 | 167 | 15.71 | 24.43 | 55.56 | 101 | 10.75 | 38.46 | 257.68 | 171 | 8.18 | 9.33 | 14.1 | 114 |
| Comoros | 26.82 | 103.71 | 286.74 | 168 | 11.98 | 18.89 | 57.61 | 132 | 19.62 | 66.67 | 239.85 | 164 | 9.49 | 13.22 | 39.28 | 47 |
| Timor-Leste | 22.97 | 99.38 | 332.7 | 169 | 6.15 | 10.83 | 76.16 | 190 | 16.19 | 60.37 | 272.98 | 166 | 4.81 | 6.88 | 42.94 | 164 |
| Gambia | 16.63 | 92.68 | 457.26 | 170 | 4.15 | 8.02 | 93.14 | 201 | 11.73 | 55.84 | 375.91 | 167 | 3.2 | 5.3 | 65.55 | 186 |
| Cabo Verde | 26.62 | 74.13 | 178.48 | 171 | 12.72 | 15.35 | 20.65 | 160 | 17.76 | 38.36 | 116.03 | 172 | 8.26 | 8.43 | 1.98 | 140 |
| Saint Lucia | 27.17 | 73.61 | 170.93 | 172 | 31.41 | 30.89 | -1.64 | 71 | 14.11 | 29.73 | 110.63 | 173 | 17.13 | 12.57 | -26.63 | 51 |
| Solomon Islands | 14.31 | 69.71 | 387.2 | 173 | 8.74 | 15.65 | 79.06 | 159 | 9.61 | 44.45 | 362.39 | 170 | 6.54 | 11.08 | 69.23 | 75 |
| Monaco | 34.01 | 63.03 | 85.36 | 174 | 60.36 | 86.05 | 42.55 | 1 | 12.23 | 17.49 | 42.98 | 181 | 18.66 | 18.88 | 1.16 | 9 |
| Andorra | 22.62 | 61.15 | 170.36 | 175 | 38.07 | 41.75 | 9.66 | 25 | 6.67 | 13.96 | 109.47 | 187 | 11.98 | 8.85 | -26.09 | 126 |
| Bermuda | 33.75 | 59.31 | 75.76 | 176 | 52.89 | 49.96 | -5.53 | 7 | 12.67 | 15.12 | 19.32 | 184 | 20.86 | 11.16 | -46.52 | 72 |
| Belize | 9.37 | 57.16 | 510.36 | 177 | 9.57 | 16.48 | 72.17 | 152 | 4.74 | 22.18 | 367.76 | 175 | 4.97 | 6.86 | 37.94 | 165 |
| Bhutan | 14.82 | 52.08 | 251.42 | 178 | 4.91 | 7.72 | 57.19 | 202 | 10.46 | 27.41 | 162.18 | 174 | 3.75 | 4.3 | 14.65 | 195 |
| Antigua and Barbuda | 16.42 | 51.45 | 213.25 | 179 | 32.1 | 46.45 | 44.69 | 11 | 7.76 | 17.65 | 127.44 | 180 | 14.66 | 16.83 | 14.78 | 19 |
| Saint Vincent and the Grenadines | 21.84 | 48.95 | 124.2 | 180 | 31.21 | 35.09 | 12.44 | 48 | 11.41 | 21.06 | 84.54 | 176 | 16.82 | 15.37 | -8.61 | 28 |
| United States Virgin Islands | 34.79 | 48.53 | 39.49 | 181 | 36.78 | 33.43 | -9.12 | 56 | 14.27 | 18.37 | 28.74 | 179 | 16.91 | 11.7 | -30.8 | 59 |
| Grenada | 18.35 | 45.06 | 145.54 | 182 | 27.97 | 39.25 | 40.35 | 33 | 10.37 | 18.65 | 79.85 | 178 | 14.98 | 17.33 | 15.66 | 14 |
| Maldives | 6.7 | 39.6 | 491.16 | 183 | 6 | 8.9 | 48.39 | 197 | 4.23 | 14.37 | 239.81 | 185 | 4.07 | 3.83 | -6.04 | 200 |
| Guam | 14.85 | 31.8 | 114.1 | 184 | 17.16 | 16.39 | -4.5 | 153 | 6.6 | 14.16 | 114.57 | 186 | 9.65 | 7.01 | -27.35 | 162 |
| Vanuatu | 7.25 | 31.01 | 327.73 | 185 | 9.51 | 15.07 | 58.5 | 163 | 4.6 | 20.17 | 338.35 | 177 | 6.9 | 10.88 | 57.79 | 79 |
| Seychelles | 7.79 | 29.51 | 278.77 | 186 | 13.88 | 23.61 | 70.1 | 108 | 4.84 | 13.59 | 180.98 | 190 | 8.65 | 11.48 | 32.75 | 65 |
| Dominica | 17.12 | 28.19 | 64.64 | 187 | 29.99 | 34.46 | 14.93 | 52 | 9.54 | 13.66 | 43.19 | 189 | 16.63 | 17.04 | 2.45 | 15 |
| Tonga | 17.24 | 28.19 | 63.56 | 188 | 28.71 | 33.78 | 17.64 | 54 | 9.89 | 16.33 | 65.13 | 182 | 17.6 | 20.12 | 14.34 | 7 |
| Samoa | 12.31 | 27.99 | 127.38 | 189 | 13.55 | 17.95 | 32.41 | 137 | 7.71 | 16.09 | 108.77 | 183 | 9.12 | 10.9 | 19.5 | 77 |
| Saint Kitts and Nevis | 13.37 | 26.41 | 97.49 | 190 | 39.32 | 36.43 | -7.34 | 42 | 8.13 | 10.77 | 32.39 | 193 | 23.53 | 16.42 | -30.22 | 21 |
| Micronesia (Federated States of) | 9.76 | 21.4 | 119.38 | 191 | 18.26 | 25.89 | 41.8 | 97 | 6.73 | 13.41 | 99.09 | 191 | 13.65 | 17.8 | 30.43 | 13 |
| Sao Tome and Principe | 5.2 | 20.8 | 299.65 | 192 | 7.98 | 15.25 | 91.12 | 161 | 3.78 | 10.93 | 189.28 | 192 | 6 | 9.02 | 50.25 | 121 |
| Kiribati | 6.92 | 19.22 | 177.76 | 193 | 16.28 | 23.53 | 44.57 | 109 | 5.05 | 13.68 | 171 | 188 | 13.08 | 18.59 | 42.13 | 11 |
| American Samoa | 5.93 | 17.47 | 194.66 | 194 | 22.08 | 34.86 | 57.86 | 50 | 3.19 | 9.84 | 208.62 | 194 | 13.97 | 20.79 | 48.87 | 4 |
| San Marino | 11.47 | 16.33 | 42.34 | 195 | 35.65 | 27.45 | -23 | 90 | 3.83 | 4.36 | 13.7 | 200 | 11.11 | 5.87 | -47.19 | 179 |
| Northern Mariana Islands | 6.37 | 15.47 | 143.06 | 196 | 22.52 | 27.89 | 23.85 | 87 | 2.47 | 7.26 | 193.72 | 195 | 12.23 | 14.58 | 19.18 | 37 |
| Greenland | 11.17 | 15.17 | 35.86 | 197 | 28.2 | 20.87 | -25.99 | 123 | 5.84 | 5.78 | -0.95 | 197 | 16.64 | 8.43 | -49.36 | 141 |
| Cook Islands | 4.67 | 11.07 | 137.03 | 198 | 34.44 | 47.22 | 37.13 | 9 | 2.39 | 5.06 | 111.98 | 198 | 19.58 | 20.62 | 5.31 | 6 |
| Marshall Islands | 2.92 | 9.6 | 228.62 | 199 | 15.06 | 22.02 | 46.24 | 120 | 1.92 | 5.97 | 211.11 | 196 | 11.31 | 15.39 | 36.06 | 27 |
| Palau | 3.78 | 7.78 | 105.72 | 200 | 34.87 | 35.4 | 1.52 | 47 | 2.15 | 4.37 | 103.29 | 199 | 22.44 | 21.9 | -2.41 | 1 |
| Tuvalu | 1.24 | 2.43 | 95.74 | 201 | 17.62 | 22.7 | 28.79 | 116 | 0.85 | 1.54 | 80.19 | 201 | 13.16 | 15.16 | 15.17 | 30 |
| Nauru | 1.26 | 2.23 | 77.02 | 202 | 20.73 | 31.6 | 52.39 | 67 | 0.75 | 1.32 | 75.96 | 202 | 13.93 | 21 | 50.7 | 3 |
| Niue | 0.51 | 0.66 | 28.87 | 203 | 23.77 | 32.59 | 37.12 | 63 | 0.35 | 0.39 | 9.86 | 203 | 15.58 | 18.79 | 20.62 | 10 |
| Tokelau | 0.27 | 0.42 | 56.88 | 204 | 21.5 | 29.49 | 37.18 | 81 | 0.18 | 0.24 | 33.39 | 204 | 14.5 | 16.6 | 14.44 | 20 |

Abbreviations: ASIR, age-standardized incidence rate; ASDR, age-standardized death rate; BC, breast cancer; UI, uncertainty interval. Data were obtained from GBD 2021. Incident cases and deaths are presented as number estimates, and ASIR and ASDR are presented per 100,000 population. Values are shown as point estimates with 95% uncertainty intervals where available. Percentage changes were calculated using point estimates between 1990 and 2021. Rankings were based on 2021 point estimates across 204 countries and territories, with rank 1 indicating the highest burden or rate.

Supplementary Table S4. Age distribution of breast cancer DALYs and deaths by SDI quintile and representative countries in 2021.

| Panel A. DALYs | |  |  |  |  |  |
| --- | --- | --- | --- | --- | --- | --- |
| Location | 15–49 years, n (95% UI) | 15–49 years, % | 50–74 years, n (95% UI) | 50–74 years, % | ≥75 years, n (95% UI) | ≥75 years, % |
| Global | 6,743,070 (6,282,867–7,237,796) | 32.7 | 11,509,826 (10,717,508–12,357,017) | 55.8 | 2,382,822 (2,035,418–2,603,946) | 11.5 |
| High SDI | 796,370 (758,335–840,524) | 18.2 | 2,519,961 (2,364,782–2,664,949) | 57.7 | 1,051,219 (856,450–1,166,899) | 24.1 |
| High-middle SDI | 1,061,906 (956,145–1,189,724) | 24.9 | 2,609,253 (2,392,213–2,900,459) | 61.2 | 591,823 (510,605–648,257) | 13.9 |
| Middle SDI | 2,229,888 (2,029,373–2,441,907) | 36.1 | 3,508,995 (3,154,167–3,904,594) | 56.9 | 430,533 (374,775–480,763) | 7 |
| Low-middle SDI | 1,837,974 (1,634,020–2,044,447) | 44.4 | 2,079,939 (1,853,332–2,311,283) | 50.3 | 217,885 (192,866–243,901) | 5.3 |
| Low SDI | 810,500 (692,295–941,577) | 48.3 | 778,805 (679,021–881,100) | 46.4 | 87,776 (78,066–98,255) | 5.2 |
| United Kingdom | 53,761 (51,607–56,241) | 16.8 | 168,732 (159,775–177,332) | 52.7 | 97,408 (82,221–106,540) | 30.4 |
| China | 883,688 (669,818–1,143,993) | 29.2 | 1,916,929 (1,495,960–2,434,205) | 63.3 | 228,788 (182,096–276,343) | 7.6 |
| India | 1,172,997 (1,011,250–1,369,123) | 42.5 | 1,427,152 (1,175,712–1,727,705) | 51.7 | 158,648 (131,217–189,860) | 5.8 |

| Panel B. Deaths | |  |  |  |  |  |
| --- | --- | --- | --- | --- | --- | --- |
| Location | 15–49 years, n (95% UI) | 15–49 years, % | 50–74 years, n (95% UI) | 50–74 years, % | ≥75 years, n (95% UI) | ≥75 years, % |
| Global | 131,024 (121,841–140,903) | 19.4 | 366,691 (343,570–392,229) | 54.4 | 176,485 (146,115–193,082) | 26.2 |
| High SDI | 14,867 (14,396–15,313) | 8.6 | 79,975 (75,305–82,980) | 46.2 | 78,422 (62,598–86,879) | 45.3 |
| High-middle SDI | 20,500 (18,340–22,864) | 13.8 | 83,751 (76,754–92,342) | 56.4 | 44,191 (36,761–48,228) | 29.8 |
| Middle SDI | 43,785 (39,877–48,115) | 23.6 | 110,573 (99,700–122,315) | 59.5 | 31,413 (26,998–35,167) | 16.9 |
| Low-middle SDI | 35,956 (32,172–39,781) | 30.4 | 66,701 (59,267–73,755) | 56.3 | 15,799 (13,940–17,626) | 13.3 |
| Low SDI | 15,790 (13,484–18,308) | 33.3 | 25,265 (22,127–28,440) | 53.3 | 6,387 (5,664–7,164) | 13.5 |
| United Kingdom | 1,010 (986–1,033) | 7.3 | 5,391 (5,146–5,555) | 38.7 | 7,513 (6,186–8,211) | 54 |
| China | 16,843 (12,654–21,852) | 18.4 | 58,580 (45,649–73,819) | 64 | 16,061 (12,695–19,487) | 17.6 |
| India | 23,041 (19,900–26,871) | 28.5 | 46,218 (38,349–55,790) | 57.2 | 11,559 (9,533–13,889) | 14.3 |

Abbreviations: BC, breast cancer; DALYs, disability-adjusted life years; SDI, socio-demographic index; UI, uncertainty interval. Percentages were calculated as age-specific numbers divided by all-age totals within each location and measure. Values are presented as number (95% UI) and percentage of total burden in 2021.

Supplementary Table S5. Age-standardized DALY rates of breast cancer attributable to selected modifiable risk factors globally and in representative countries, 1990 and 2021.

| Location | Risk factor | 1990 rate, per 100,000 (95% UI) | 2021 rate, per 100,000 (95% UI) | Change 1990–2021, % |
| --- | --- | --- | --- | --- |
| Global | Diet high in red meat | 33.31 (-0.01–71.68) | 28.37 (-0.01–60.54) | -14.8 |
| Global | High body-mass index | 11.19 (-0.41–22.71) | 11.63 (-0.49–22.68) | 3.9 |
| Global | High fasting plasma glucose | 7.33 (-2.13–17.25) | 9.43 (-2.77–22.26) | 28.8 |
| Global | High alcohol use | 11.68 (8.50–15.26) | 6.75 (4.78–8.95) | -42.2 |
| Global | Tobacco | 10.91 (4.62–17.19) | 6.46 (2.12–10.90) | -40.8 |
| Global | Low physical activity | 5.47 (1.10–9.50) | 4.81 (0.97–8.45) | -11.9 |
| United Kingdom | Diet high in red meat | 78.76 (-0.03–167.41) | 38.85 (-0.01–82.44) | -50.7 |
| United Kingdom | High body-mass index | 31.25 (-1.33–63.60) | 19.35 (-0.78–37.66) | -38.1 |
| United Kingdom | High fasting plasma glucose | 11.53 (-3.15–27.90) | 9.60 (-2.45–23.42) | -16.7 |
| United Kingdom | High alcohol use | 46.75 (33.29–59.90) | 24.57 (17.15–31.88) | -47.4 |
| United Kingdom | Tobacco | 40.91 (27.14–54.65) | 12.90 (8.59–17.58) | -68.5 |
| United Kingdom | Low physical activity | 21.49 (4.29–37.87) | 10.93 (2.12–18.98) | -49.1 |
| China | Diet high in red meat | 20.49 (-0.01–45.17) | 19.95 (-0.01–44.22) | -2.6 |
| China | High body-mass index | 3.56 (-0.10–7.43) | 7.31 (-0.24–15.66) | 105 |
| China | High fasting plasma glucose | 4.15 (-1.22–10.37) | 5.05 (-1.60–12.23) | 21.7 |
| China | High alcohol use | 1.82 (1.03–2.80) | 1.96 (1.20–2.93) | 7.9 |
| China | Tobacco | 5.29 (0.27–10.52) | 4.35 (0.06–9.01) | -17.8 |
| China | Low physical activity | 2.56 (0.50–4.76) | 2.61 (0.47–4.91) | 1.9 |
| India | Diet high in red meat | 6.47 (-0.00–14.67) | 10.07 (-0.00–23.21) | 55.5 |
| India | High body-mass index | 0.82 (-0.29–2.04) | 3.92 (-0.46–8.40) | 378.3 |
| India | High fasting plasma glucose | 4.00 (-1.20–9.37) | 8.68 (-2.28–21.24) | 117.2 |
| India | High alcohol use | 0.44 (0.11–0.72) | 0.91 (0.52–1.39) | 105.3 |
| India | Tobacco | 3.52 (0.13–6.98) | 3.43 (0.27–7.02) | -2.4 |
| India | Low physical activity | 2.33 (0.48–4.23) | 3.30 (0.69–6.16) | 41.5 |

Abbreviations: BMI, body mass index; BC, breast cancer; DALYs, disability-adjusted life years; FPG, fasting plasma glucose; SDI, socio-demographic index; UI, uncertainty interval. Data were obtained from GBD 2021. Rates are age-standardized DALY rates attributable to selected modifiable risk factors and are presented per 100,000 population. Values are shown as point estimates with 95% uncertainty intervals. Percentage changes were calculated using point estimates. The years 1990, 2015, and 2021 were selected to summarize long-term changes and recent shifts in risk-attributable burden. Risk-attributable DALYs were estimated within the GBD comparative risk assessment framework and should be interpreted as population-level attributable burden rather than evidence of individual-level causality.

Supplementary Table S6. Age-specific proportions of breast cancer deaths and disability-adjusted life years attributable to selected risk factors in China, the United Kingdom, and India, 1990 and 2021

| Measure | Country | Age category | 1990 | | | | | | |  | 2021 | | | | | | |
| --- | --- | --- | --- | --- | --- | --- | --- | --- | --- | --- | --- | --- | --- | --- | --- | --- | --- |
|  |  |  | Diet high in red meat | High alcohol use | Tobacco | | Low physical activity | High body-mass index | High fasting plasma glucose |  | Diet high in red meat | High alcohol use | Tobacco | | Low physical activity | High body-mass index | High fasting plasma glucose |
| Deaths | Global | 15–49 years | 11.99 (0.00 to 25.70) | 4.70 (3.53 to 5.95) | | 4.43 (1.55 to 7.29) | 1.31 (0.27 to 2.27) | −3.15 (−6.87 to 0.00) | 1.53 (−0.44 to 3.73) |  | 11.24 (0.00 to 23.85) | 2.68 (1.91 to 3.50) | 2.57 (0.54 to 4.65) | 1.33 (0.27 to 2.33) | | −3.73 (−8.35 to 0.00) | 2.36 (−0.72 to 5.47) |
| Deaths | Global | 50–74 years | 12.71 (0.00 to 27.07) | 4.36 (2.96 to 6.01) | | 4.25 (1.87 to 6.61) | 2.19 (0.45 to 3.78) | 7.81 (−0.22 to 15.56) | 3.26 (−0.96 to 7.72) |  | 12.06 (0.00 to 25.84) | 2.82 (1.93 to 3.90) | 2.92 (1.08 to 4.76) | 2.20 (0.44 to 3.81) | | 9.36 (−0.28 to 18.46) | 4.73 (−1.37 to 11.19) |
| Deaths | Global | ≥75 years | 13.21 (0.00 to 28.02) | 3.42 (1.46 to 5.22) | | 2.53 (1.13 to 3.92) | 3.49 (0.69 to 6.16) | 7.36 (−0.21 to 14.83) | 4.01 (−1.15 to 9.36) |  | 12.70 (0.00 to 27.07) | 2.79 (1.76 to 4.08) | 1.86 (0.73 to 2.96) | 3.49 (0.73 to 6.13) | | 8.62 (−0.25 to 17.36) | 5.74 (−1.63 to 13.58) |
| Deaths | Global | All ages | 12.67 (0.00 to 27.01) | 4.21 (2.97 to 5.70) | | 3.88 (1.63 to 6.12) | 2.30 (0.47 to 3.98) | 5.27 (−0.17 to 10.33) | 3.05 (−0.90 to 7.22) |  | 12.07 (0.00 to 25.79) | 2.78 (1.93 to 3.80) | 2.57 (0.90 to 4.25) | 2.37 (0.49 to 4.14) | | 6.62 (−0.22 to 12.97) | 4.53 (−1.30 to 10.67) |
| Deaths | Global | Age-standardized | 12.75 (0.00 to 27.17) | 4.12 (2.85 to 5.65) | | 3.73 (1.59 to 5.85) | 2.46 (0.50 to 4.27) | 5.62 (−0.18 to 11.05) | 3.17 (−0.93 to 7.47) |  | 12.08 (0.00 to 25.80) | 2.78 (1.94 to 3.80) | 2.54 (0.88 to 4.20) | 2.40 (0.49 to 4.19) | | 6.54 (−0.22 to 12.82) | 4.53 (−1.30 to 10.68) |
| Deaths | United Kingdom | 15–49 years | 13.62 (−0.00 to 28.85) | 11.52 (8.35 to 14.89) | | 8.28 (5.18 to 11.32) | 3.01 (0.60 to 5.46) | −5.35 (−11.81 to −0.00) | 0.99 (−0.27 to 2.30) |  | 13.60 (−0.01 to 28.81) | 11.50 (7.84 to 15.12) | 4.89 (2.96 to 6.80) | 3.06 (0.55 to 5.56) | | −6.29 (−13.94 to −0.01) | 1.56 (−0.45 to 3.72) |
| Deaths | United Kingdom | 50–74 years | 13.66 (−0.01 to 28.92) | 6.89 (4.56 to 9.72) | | 7.14 (4.87 to 9.43) | 3.91 (0.81 to 6.82) | 9.75 (−0.29 to 19.67) | 2.39 (−0.66 to 5.72) |  | 13.60 (−0.00 to 28.74) | 8.02 (5.53 to 10.85) | 4.86 (3.24 to 6.44) | 3.95 (0.78 to 6.88) | | 11.76 (−0.37 to 23.06) | 3.98 (−1.02 to 9.68) |
| Deaths | United Kingdom | ≥75 years | 13.56 (−0.01 to 28.74) | 4.38 (1.52 to 6.94) | | 3.97 (2.57 to 5.41) | 4.78 (0.94 to 8.68) | 8.42 (−0.25 to 17.03) | 3.09 (−0.82 to 7.39) |  | 13.42 (−0.00 to 28.61) | 4.83 (2.89 to 7.20) | 2.71 (1.79 to 3.79) | 4.83 (0.98 to 8.53) | | 10.36 (−0.33 to 20.80) | 4.86 (−1.25 to 11.94) |
| Deaths | United Kingdom | All ages | 13.62 (−0.01 to 28.84) | 6.39 (4.26 to 8.81) | | 5.99 (4.01 to 7.96) | 4.16 (0.85 to 7.36) | 7.57 (−0.24 to 15.37) | 2.52 (−0.68 to 6.04) |  | 13.50 (−0.00 to 28.71) | 6.55 (4.49 to 9.01) | 3.70 (2.47 to 4.97) | 4.36 (0.87 to 7.54) | | 9.69 (−0.32 to 19.24) | 4.28 (−1.10 to 10.63) |
| Deaths | United Kingdom | Age-standardized | 13.62 (−0.01 to 28.83) | 6.86 (4.70 to 9.26) | | 6.27 (4.18 to 8.39) | 4.04 (0.82 to 7.14) | 7.02 (−0.23 to 14.27) | 2.35 (−0.64 to 5.63) |  | 13.53 (−0.00 to 28.72) | 7.22 (5.03 to 9.64) | 3.98 (2.66 to 5.35) | 4.18 (0.83 to 7.22) | | 8.79 (−0.30 to 17.46) | 3.98 (−1.03 to 9.83) |
| Deaths | China | 15–49 years | 13.33 (−0.00 to 28.32) | 1.22 (0.72 to 1.75) | | 3.19 (−0.22 to 6.58) | 1.09 (0.22 to 2.16) | 0.00 (0.00 to 0.00) | 1.62 (−0.47 to 3.98) |  | 13.58 (−0.01 to 28.70) | 1.40 (0.86 to 2.08) | 2.96 (−0.21 to 6.19) | 1.22 (0.20 to 2.43) | | 0.00 (0.00 to 0.00) | 2.43 (−0.72 to 5.79) |
| Deaths | China | 50–74 years | 13.55 (−0.01 to 28.71) | 1.25 (0.69 to 1.88) | | 3.77 (0.48 to 7.01) | 1.93 (0.39 to 3.56) | 4.10 (−0.11 to 8.22) | 3.46 (−1.04 to 8.28) |  | 13.67 (−0.01 to 28.88) | 1.37 (0.80 to 2.05) | 3.03 (0.20 to 5.83) | 1.98 (0.37 to 3.57) | | 8.00 (−0.23 to 16.08) | 4.08 (−1.19 to 9.66) |
| Deaths | China | ≥75 years | 13.36 (−0.00 to 28.26) | 0.79 (0.36 to 1.33) | | 3.45 (0.57 to 6.24) | 3.36 (0.69 to 6.28) | 3.22 (−0.09 to 6.42) | 4.30 (−1.28 to 10.08) |  | 13.63 (−0.00 to 28.88) | 0.81 (0.40 to 1.27) | 2.70 (0.23 to 5.12) | 3.45 (0.72 to 6.32) | | 6.29 (−0.17 to 12.67) | 4.64 (−1.35 to 10.65) |
| Deaths | China | All ages | 13.45 (−0.00 to 28.53) | 1.19 (0.69 to 1.74) | | 3.54 (0.23 to 6.74) | 1.81 (0.38 to 3.26) | 2.63 (−0.07 to 5.30) | 2.94 (−0.88 to 6.95) |  | 13.65 (−0.01 to 28.85) | 1.28 (0.78 to 1.88) | 2.96 (0.16 to 5.75) | 2.10 (0.40 to 3.66) | | 6.23 (−0.18 to 12.57) | 3.88 (−1.12 to 9.11) |
| Deaths | China | Age-standardized | 13.47 (−0.00 to 28.56) | 1.13 (0.64 to 1.68) | | 3.56 (0.32 to 6.70) | 2.07 (0.43 to 3.68) | 2.81 (−0.08 to 5.67) | 3.17 (−0.94 to 7.49) |  | 13.62 (−0.01 to 28.79) | 1.25 (0.77 to 1.84) | 2.93 (0.15 to 5.70) | 2.13 (0.41 to 3.69) | | 5.82 (−0.17 to 11.72) | 3.81 (−1.09 to 8.98) |
| Deaths | India | 15–49 years | 4.17 (−0.00 to 9.56) | 0.34 (0.06 to 0.57) | | 2.28 (−0.07 to 4.64) | 0.94 (0.20 to 1.78) | −2.26 (−4.70 to −0.00) | 1.84 (−0.54 to 4.44) |  | 4.43 (−0.00 to 9.73) | 0.49 (0.28 to 0.75) | 1.43 (−0.11 to 3.02) | 0.84 (0.16 to 1.62) | | −3.20 (−7.03 to −0.00) | 2.70 (−0.80 to 6.23) |
| Deaths | India | 50–74 years | 4.92 (−0.00 to 10.92) | 0.31 (0.08 to 0.54) | | 2.68 (0.23 to 5.07) | 2.00 (0.39 to 3.59) | 2.70 (−0.07 to 5.39) | 3.45 (−1.00 to 8.19) |  | 5.22 (−0.00 to 11.31) | 0.43 (0.22 to 0.69) | 1.83 (0.22 to 3.42) | 1.89 (0.39 to 3.46) | | 5.11 (−0.17 to 10.18) | 5.11 (−1.49 to 12.09) |
| Deaths | India | ≥75 years | 5.29 (−0.00 to 11.72) | 0.18 (0.05 to 0.32) | | 2.48 (0.03 to 4.82) | 3.82 (0.80 to 6.75) | 1.89 (−0.05 to 3.76) | 4.70 (−1.26 to 11.30) |  | 5.38 (−0.00 to 12.02) | 0.26 (0.12 to 0.43) | 1.74 (0.22 to 3.37) | 3.83 (0.79 to 6.81) | | 3.58 (−0.12 to 7.53) | 6.21 (−1.71 to 14.89) |
| Deaths | India | All ages | 4.66 (−0.00 to 10.28) | 0.31 (0.08 to 0.51) | | 2.52 (0.10 to 4.90) | 1.74 (0.36 to 3.05) | 0.77 (−0.16 to 1.73) | 2.93 (−0.85 to 6.93) |  | 5.01 (−0.00 to 10.88) | 0.43 (0.24 to 0.65) | 1.70 (0.17 to 3.26) | 1.87 (0.39 to 3.40) | | 2.52 (−0.15 to 5.13) | 4.58 (−1.31 to 10.77) |
| Deaths | India | Age-standardized | 4.81 (−0.00 to 10.65) | 0.29 (0.07 to 0.48) | | 2.54 (0.12 to 4.90) | 2.08 (0.43 to 3.69) | 1.14 (−0.13 to 2.37) | 3.25 (−0.95 to 7.71) |  | 5.08 (−0.00 to 11.03) | 0.41 (0.23 to 0.63) | 1.72 (0.19 to 3.27) | 2.08 (0.43 to 3.78) | | 2.79 (−0.12 to 5.78) | 4.78 (−1.37 to 11.25) |
| DALYs | Global | 15–49 years | 11.94 (0.00 to 25.60) | 4.73 (3.55 to 5.97) | | 4.38 (1.53 to 7.21) | 1.29 (0.27 to 2.23) | −3.11 (−6.77 to 0.00) | 1.49 (−0.43 to 3.61) |  | 11.20 (0.00 to 23.76) | 2.72 (1.93 to 3.54) | 2.54 (0.53 to 4.61) | 1.31 (0.26 to 2.29) | | −3.70 (−8.28 to 0.00) | 2.30 (−0.70 to 5.32) |
| DALYs | Global | 50–74 years | 12.70 (0.00 to 27.06) | 4.47 (3.08 to 6.10) | | 4.36 (1.90 to 6.81) | 2.11 (0.43 to 3.65) | 7.81 (−0.22 to 15.50) | 3.15 (−0.94 to 7.46) |  | 12.08 (0.00 to 25.89) | 2.89 (1.99 to 3.95) | 3.00 (1.10 to 4.92) | 2.13 (0.43 to 3.71) | | 9.40 (−0.29 to 18.50) | 4.57 (−1.33 to 10.80) |
| DALYs | Global | ≥75 years | 13.22 (0.00 to 28.04) | 3.47 (1.52 to 5.28) | | 2.58 (1.17 to 3.97) | 3.44 (0.68 to 6.07) | 7.46 (−0.21 to 15.02) | 4.05 (−1.16 to 9.46) |  | 12.70 (0.00 to 27.08) | 2.82 (1.83 to 4.14) | 1.92 (0.77 to 3.02) | 3.44 (0.72 to 6.04) | | 8.71 (−0.25 to 17.54) | 5.82 (−1.65 to 13.76) |
| DALYs | Global | All ages | 12.48 (0.00 to 26.66) | 4.46 (3.25 to 5.81) | | 4.18 (1.69 to 6.66) | 1.96 (0.40 to 3.40) | 3.84 (−0.18 to 7.49) | 2.65 (−0.78 to 6.30) |  | 11.86 (0.00 to 25.33) | 2.83 (2.00 to 3.79) | 2.73 (0.89 to 4.60) | 2.01 (0.41 to 3.52) | | 5.04 (−0.19 to 9.81) | 3.97 (−1.15 to 9.35) |
| DALYs | Global | Age-standardized | 12.56 (0.00 to 26.81) | 4.41 (3.20 to 5.79) | | 4.12 (1.69 to 6.53) | 2.06 (0.42 to 3.57) | 4.22 (−0.15 to 8.27) | 2.76 (−0.82 to 6.56) |  | 11.85 (0.00 to 25.30) | 2.82 (2.00 to 3.78) | 2.70 (0.87 to 4.56) | 2.01 (0.41 to 3.52) | | 4.86 (−0.20 to 9.44) | 3.94 (−1.14 to 9.28) |
| DALYs | United Kingdom | 15–49 years | 13.60 (−0.00 to 28.81) | 11.56 (8.42 to 14.93) | | 8.21 (5.14 to 11.22) | 2.99 (0.60 to 5.39) | −5.32 (−11.73 to −0.00) | 0.97 (−0.27 to 2.26) |  | 13.58 (−0.01 to 28.77) | 11.51 (7.86 to 15.09) | 4.81 (2.92 to 6.69) | 3.04 (0.55 to 5.48) | | −6.25 (−13.85 to −0.01) | 1.52 (−0.44 to 3.63) |
| DALYs | United Kingdom | 50–74 years | 13.66 (−0.01 to 28.92) | 7.19 (4.90 to 9.96) | | 7.28 (4.94 to 9.66) | 3.84 (0.79 to 6.71) | 9.74 (−0.29 to 19.66) | 2.28 (−0.63 to 5.46) |  | 13.60 (−0.00 to 28.79) | 8.35 (5.79 to 11.13) | 4.97 (3.29 to 6.60) | 3.87 (0.76 to 6.74) | | 11.75 (−0.37 to 23.05) | 3.77 (−0.97 to 9.15) |
| DALYs | United Kingdom | ≥75 years | 13.58 (−0.00 to 28.79) | 4.46 (1.53 to 7.01) | | 4.11 (2.70 to 5.57) | 4.75 (0.95 to 8.62) | 8.51 (−0.25 to 17.15) | 3.10 (−0.82 to 7.46) |  | 13.44 (−0.00 to 28.67) | 4.97 (3.03 to 7.44) | 2.87 (1.92 to 3.94) | 4.80 (0.99 to 8.40) | | 10.47 (−0.33 to 20.98) | 4.92 (−1.27 to 12.19) |
| DALYs | United Kingdom | All ages | 13.63 (−0.01 to 28.87) | 7.55 (5.37 to 9.91) | | 6.80 (4.52 to 9.11) | 3.85 (0.78 to 6.79) | 6.21 (−0.22 to 12.47) | 2.17 (−0.59 to 5.20) |  | 13.55 (−0.00 to 28.73) | 7.85 (5.52 to 10.35) | 4.30 (2.86 to 5.73) | 4.02 (0.79 to 6.92) | | 8.33 (−0.30 to 16.53) | 3.74 (−0.97 to 9.17) |
| DALYs | United Kingdom | Age-standardized | 13.63 (−0.01 to 28.86) | 8.09 (5.84 to 10.34) | | 7.08 (4.67 to 9.52) | 3.72 (0.75 to 6.49) | 5.41 (−0.23 to 10.87) | 2.00 (−0.55 to 4.76) |  | 13.56 (−0.00 to 28.74) | 8.58 (6.03 to 11.08) | 4.51 (2.98 to 5.99) | 3.82 (0.74 to 6.55) | | 6.76 (−0.28 to 13.25) | 3.36 (−0.87 to 8.15) |
| DALYs | China | 15–49 years | 13.22 (−0.00 to 28.10) | 1.21 (0.72 to 1.73) | | 3.14 (−0.24 to 6.49) | 1.08 (0.22 to 2.10) | 0.00 (0.00 to 0.00) | 1.57 (−0.46 to 3.89) |  | 13.55 (−0.01 to 28.62) | 1.40 (0.87 to 2.08) | 2.94 (−0.22 to 6.14) | 1.21 (0.21 to 2.39) | | 0.00 (0.00 to 0.00) | 2.38 (−0.70 to 5.69) |
| DALYs | China | 50–74 years | 13.55 (−0.01 to 28.74) | 1.26 (0.71 to 1.89) | | 3.76 (0.42 to 7.01) | 1.84 (0.38 to 3.39) | 4.14 (−0.11 to 8.27) | 3.35 (−1.02 to 8.03) |  | 13.67 (−0.01 to 28.88) | 1.39 (0.82 to 2.09) | 3.07 (0.16 to 5.90) | 1.90 (0.35 to 3.39) | | 8.05 (−0.23 to 16.16) | 3.99 (−1.16 to 9.47) |
| DALYs | China | ≥75 years | 13.37 (−0.00 to 28.21) | 0.80 (0.36 to 1.35) | | 3.43 (0.55 to 6.27) | 3.31 (0.66 to 6.25) | 3.26 (−0.09 to 6.46) | 4.32 (−1.28 to 10.10) |  | 13.64 (−0.00 to 28.91) | 0.84 (0.42 to 1.34) | 2.72 (0.26 to 5.24) | 3.40 (0.70 to 6.24) | | 6.38 (−0.18 to 12.79) | 4.67 (−1.37 to 10.73) |
| DALYs | China | All ages | 13.39 (−0.00 to 28.42) | 1.22 (0.72 to 1.76) | | 3.45 (0.10 to 6.70) | 1.55 (0.32 to 2.77) | 2.14 (−0.06 to 4.30) | 2.55 (−0.77 to 6.02) |  | 13.63 (−0.01 to 28.80) | 1.35 (0.82 to 1.99) | 3.00 (0.07 to 5.91) | 1.81 (0.34 to 3.14) | | 5.58 (−0.16 to 11.23) | 3.57 (−1.03 to 8.33) |
| DALYs | China | Age-standardized | 13.45 (−0.00 to 28.54) | 1.20 (0.70 to 1.75) | | 3.50 (0.16 to 6.73) | 1.69 (0.35 to 3.02) | 2.34 (−0.06 to 4.72) | 2.73 (−0.82 to 6.45) |  | 13.59 (−0.01 to 28.70) | 1.35 (0.83 to 1.97) | 2.97 (0.04 to 5.87) | 1.78 (0.33 to 3.08) | | 4.99 (−0.14 to 10.02) | 3.44 (−0.99 to 8.02) |
| DALYs | India | 15–49 years | 4.09 (−0.00 to 9.38) | 0.34 (0.06 to 0.56) | | 2.23 (−0.09 to 4.57) | 0.91 (0.19 to 1.70) | −2.21 (−4.62 to −0.00) | 1.77 (−0.53 to 4.24) |  | 4.34 (−0.00 to 9.56) | 0.49 (0.28 to 0.74) | 1.40 (−0.11 to 2.96) | 0.81 (0.16 to 1.56) | | −3.14 (−6.86 to −0.00) | 2.60 (−0.77 to 5.99) |
| DALYs | India | 50–74 years | 4.85 (−0.00 to 10.79) | 0.32 (0.08 to 0.55) | | 2.69 (0.24 to 5.13) | 1.88 (0.36 to 3.51) | 2.76 (−0.07 to 5.50) | 3.36 (−0.97 to 8.00) |  | 5.16 (−0.00 to 11.19) | 0.44 (0.22 to 0.71) | 1.83 (0.21 to 3.44) | 1.77 (0.38 to 3.28) | | 5.22 (−0.17 to 10.42) | 4.97 (−1.46 to 11.74) |
| DALYs | India | ≥75 years | 5.32 (−0.00 to 11.85) | 0.19 (0.05 to 0.34) | | 2.48 (0.04 to 4.81) | 3.78 (0.79 to 6.72) | 1.93 (−0.06 to 3.91) | 4.66 (−1.25 to 11.20) |  | 5.40 (−0.00 to 12.09) | 0.28 (0.12 to 0.45) | 1.73 (0.23 to 3.34) | 3.79 (0.78 to 6.76) | | 3.66 (−0.12 to 7.73) | 6.23 (−1.70 to 15.04) |
| DALYs | India | All ages | 4.48 (−0.00 to 9.88) | 0.33 (0.07 to 0.53) | | 2.45 (0.06 to 4.82) | 1.44 (0.30 to 2.54) | 0.21 (−0.43 to 0.90) | 2.59 (−0.75 to 6.08) |  | 4.82 (−0.00 to 10.38) | 0.45 (0.26 to 0.68) | 1.64 (0.10 to 3.19) | 1.48 (0.31 to 2.71) | | 1.58 (−0.22 to 3.34) | 4.04 (−1.17 to 9.41) |
| DALYs | India | Age-standardized | 4.61 (−0.00 to 10.15) | 0.32 (0.08 to 0.52) | | 2.50 (0.09 to 4.88) | 1.66 (0.34 to 2.89) | 0.58 (−0.20 to 1.45) | 2.83 (−0.82 to 6.69) |  | 4.88 (−0.00 to 10.53) | 0.44 (0.25 to 0.67) | 1.66 (0.12 to 3.20) | 1.60 (0.33 to 2.93) | | 1.90 (−0.21 to 3.84) | 4.21 (−1.22 to 9.86) |

Footnote: Data are presented as percentage (95% uncertainty interval) of total breast cancer deaths or DALYs attributable to each risk factor. Estimates are for both sexes combined. Percentages for individual risk factors should not be summed because attributable burdens may overlap and are estimated separately within the GBD comparative risk assessment framework. Negative estimates or uncertainty bounds reflect modeled inverse associations or estimation uncertainty for specific age–risk combinations and should be interpreted within the GBD risk framework. Abbreviations: BMI, body-mass index; DALYs, disability-adjusted life years; FPG, fasting plasma glucose; GBD, Global Burden of Disease; UI, uncertainty interval.
